# The oxidized thiol proteome in aging and cataractous mouse and human lens revealed by ICAT labeling

**DOI:** 10.1111/acel.12548

**Published:** 2016-11-13

**Authors:** Benlian Wang, Grant Hom, Sheng Zhou, Minfei Guo, Binbin Li, Jing Yang, Vincent M. Monnier, Xingjun Fan

**Affiliations:** ^1^ Center for ProteomicsCase Western Reserve UniversityClevelandOH44120USA; ^2^ Department of PathologyCase Western Reserve UniversityClevelandOH44120USA; ^3^ State Key Laboratory of OphthalmologyZhongshan Ophthalmic CenterSun Yat‐sen UniversityGuangzhouChina; ^4^ Department of OphthalmologyThe Huichang County People's HospitalJiangxiChina; ^5^ Department of OphthalmologyGanzhou City People's HospitalJiangxiChina; ^6^ Department of BiochemistryCase Western Reserve UniversityClevelandOH44120USA

**Keywords:** aging, cataractogenesis, disulfide, mass spectrometry, proteomics, reactive oxygen species

## Abstract

Age‐related cataractogenesis is associated with disulfide‐linked high molecular weight (HMW) crystallin aggregates. We recently found that the lens crystallin disulfidome was evolutionarily conserved in human and glutathione‐depleted mouse (LEGSKO) cataracts and that it could be mimicked by oxidation *in vitro* (*Mol. Cell Proteomics*,** 14, 3211‐23 (2015)). To obtain a comprehensive blueprint of the oxidized key regulatory and cytoskeletal proteins underlying cataractogenesis, we have now used the same approach to determine, in the same specimens, all the disulfide‐forming noncrystallin proteins identified by ICAT proteomics. Seventy‐four, 50, and 54 disulfide‐forming proteins were identified in the human and mouse cataracts and the *in vitro* oxidation model, respectively, of which 17 were common to all three groups. Enzymes with oxidized cysteine at critical sites include GAPDH (hGAPDH, Cys247), glutathione synthase (hGSS, Cys294), aldehyde dehydrogenase (hALDH1A1, Cys126 and Cys186), sorbitol dehydrogenase (hSORD, Cys140, Cys165, and Cys179), and PARK7 (hPARK7, Cys46 and Cys53). Extensive oxidation was also present in lens‐specific intermediate filament proteins, such as BFSP1 and BFSP12 (hBFSP1 and hBFSP12, Cys167, Cys65, and Cys326), vimentin (mVim, Cys328), and cytokeratins, as well as microfilament and microtubule filament proteins, such as tubulin and actins. While the biological impact of these modifications for lens physiology remains to be determined, many of these oxidation sites have already been associated with either impaired metabolism or cytoskeletal architecture, strongly suggesting that they have a pathogenic role in cataractogenesis. By extrapolation, these findings may be of broader significance for age‐ and disease‐related dysfunctions associated with oxidant stress.**

**Abbreviations**
DTPA: diethylene triamine pentaacetic acid
Gclc: γ‐glutamyl cysteine ligase catalytic subunit
GSH: glutathione
HMW: high molecular weight
IAM: iodoacetamide
ICAT: isotope‐coded affinity tag
LOCS: Lens Opacities Classification System
RCS: reactive carbonyls
ROS: reactive oxygen species
TCA: trichloroacetic acid
TCEP: tris (2‐carboxyethyl) phosphine
TFA: trifluoric acid

## Introduction

The mammalian genome encodes 214 000 cysteine residues (Go & Jones, 2013). Some of the cysteine residue pairs are playing a key role in high‐ordered protein structure and conformation through intradisulfide bonds (Borges & Sherma, 2014). These cysteine pairs tend to be evolutionarily conserved (Thornton, 1981), and any disturbance of this precise disulfide bond formation will likely have a detrimental effect on protein function. Many of the free cysteine residues are also actively involved in redox signaling. It has been estimated that 10–20% of cysteine residues are redox‐active (Go & Jones, 2013). These cysteine residues, together with other redox regulatory components such as glutathione (GSH), oxidized glutathione (GSSG), cysteine, thioredoxin (Trx), and thioredoxin reductase (TrxR), constitute a powerful force in maintaining intracellular and extracellular redox status. Oxidation of these cysteine residues will give rise to an imbalance between intra‐ or extracellular redox state, which may trigger regulatory gene expression and alter protein stability (Furukawa *et al*., 2006). Finally, a large number of cysteine residues are not directly involved in protein folding and redox signaling, but can have a profound impact on protein conformation and function when becoming oxidized (Rogers *et al*., 1996). Over the years, several groundbreaking methods and techniques have offered researchers great opportunities to systematically study protein cysteine oxidation, including cysteine sulfenylation, sulfinylation, sulfonylation, nitrosylation, and disulfide formation (Paulsen & Carroll, 2013).

The present study constitutes a logical extension of a recent study in which we have systematically mapped the ‘disulfidome’ of crystallins from the aging and cataractous human lens and found surprisingly conserved oxidation sites between human age‐related cataracts, the LEGSKO mouse model of age‐related cataract linked to glutathione depletion, and oxidative changes in mouse lens homogenate exposed to H_2_O_2_ *in vitro* (Fan *et al*., 2015). In a nutshell, cysteine oxidation into disulfides was highly conserved in the β‐crystallin family, but only partly conserved in the γ‐crystallin family, implying complex *in vivo* unfolding mechanisms underlying the oxidation of sites not readily accessible to H_2_O_2_ and other cellular oxidants. While these results brought invaluable information for the potential links between *de novo* intra‐ and intermolecular disulfide formation and impaired crystallin structure and assembly, they did not provide information on the quantitatively minor but functionally critical noncrystallin proteins involved in controlling the redox state, overall metabolism, and cytoskeleton functions of the lens.

Previous studies by others have shed partial light on the presence and potential roles of noncrystallin oxidation sites in human and rodent models of cataracts. Padgaonkar *et al*. (1999) reported a significant loss of lens cytoskeletal protein in guinea pigs after hyperbaric oxygen (HBO) treatment and proposed that the loss of thiol group was due to disulfide cross‐linking. In another study (Padgaonkar *et al*., 1989), they found over 95% of glyceraldehyde‐3 phosphate dehydrogenase (GAPDH) activity was depleted in rabbit lens after 24‐h HBO treatment. In a later study, Yan *et al*. (2006) found GAPDH activity in aged and cataractous lens can be revived by thioredoxin (Trx) and thioredoxin reductase (TrxR) further supporting the notion that disulfide bond formation may be responsible for the inactivation of the enzyme. Disulfide exchange has been indicated as playing a key role in aldose reductase activity, and the formation of mixed disulfides has also been found in the lens (Mizoguchi *et al*., 1999). Lou MF *et al*. (Lou & Dickerson, 1992) have reported that protein‐thiol mixed disulfides generally increase with aging in the human lens.

While each of these studies provided valuable information, then available techniques did not allow researcher to systematically identify and quantitate abnormal cysteine disulfide‐forming target proteins. ICAT is a newly developed approach to tackle this problem. ICAT reagent contains a biotinylated tag with high selectivity and reversible affinity (Shiio & Aebersold, 2006). Using affinity purification through avidin‐agarose beads, one can enrich only the peptides with disulfide bond. This enormously simplifies probing the huge pool of a mixture of peptides from lens digest. More importantly, the relative extent of cysteine oxidation can be easily estimated by simple comparison of the integrated peak intensities in the doublets corresponding to the heavy and the light ICAT labels. For this reason, we mined the ICAT labeling data that we recently deposited into the ProteomeXchange Consortium, for the purpose of identifying (i) all noncrystallin disulfides that are common to human and LEGSKO mouse cataracts and in their *in vitro* oxidation, (ii) the sites and patterns of these cysteine residues that are oxidized during aging and oxidation, and (iii) the potential impact on lens biology based on their molecular functions. By mapping the cysteine residues oxidation sites and patterns *in vitro* and *in vivo*, we hope to better understand the significance of the sulfhydryl groups in lens biology as they have also been linked to various diseases other than cataractogenesis (Grek & Townsend, 2014).

Below we report that mining the oxidative proteome of the human and LEGSKO mouse cataractous lens has helped us to uncover and identify extensive disulfide formation sites in cysteine residues that are critical for the proper function of key cellular proteins involved in lens metabolism, such as GAPDH, redox homeostasis, such as peroxiredoxin 6 (PRDX6), and cytoskeletal structure, such as phakinin (BFSP2) and filensin (BFSP1).

## Results

**Figure 1:**
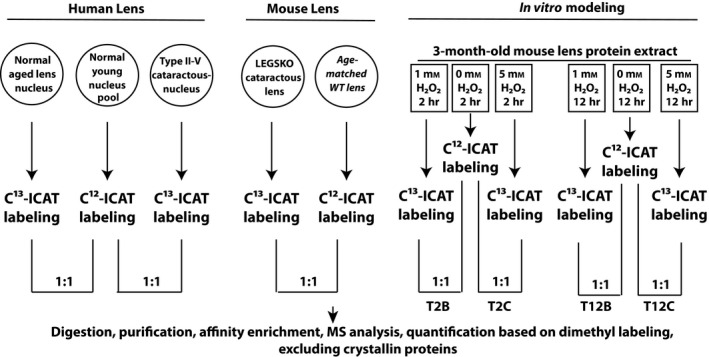
Summary of the experimental design and analytical procedures that are used in this study. For *in vitro* modeling experiment, the sample identifications (T2B, T2C, T12B, and T12C) are listed under each oxidation condition.

Three sets of data were collected in this study as illustrated in Fig. 1. They constitute a map of the cysteine oxidation sites detected in aged normal and aged cataractous human lens, GSH‐deficient mouse (LEGSKO mouse) lens, and the *in vitro* modeling of the mouse lens protein extract oxidized with 1 and 5 mm hydrogen peroxide (H_2_O_2_) for up to 12 h. It is important to understand that each peptide reported below represents the ratio of the peptide labeled with the heavy chain (C^13^ ICAT stable isotope) that is present in either aged normal human lens, aged cataractous human lens, LEGSKO or hydrogen peroxide‐oxidized mouse lens protein extract, vs. the identical peptide labeled with the light chain (C^12^ ICAT isotope) present in extract from young human lens, age‐matched WT mouse lens, and mouse lens extract without hydrogen peroxide oxidation. Therefore, the ratio of ICAT heavy/ICAT light reflects the relative degree of disulfide bonding occurring in the aged normal and aged cataractous human lens peptides vs. the degree of disulfide bonding occurring in the same peptides from young control lenses. The same principle applies to LEGSKO vs. age‐matched control and H_2_O_2_‐oxidized vs. nonoxidized control. The concept of how ICAT labeling and dimethyl labeling are used to quantitatively determine disulfide bond is illustrated in Fig. S1 (Supporting information), and the typical mass spectrum of ICAT and dimethyl labeling is shown in Figs S2 and S3 (Supporting information). All the MS data, excluding crystallins, were quantitatively analyzed after adjustment with dimethyl labeling to compare levels of the individual protein. We only report those proteins that are both involved in disulfide formation and for which both deuterated and nondeuterated dimethyl‐labeled peptides could be identified based on Mascot ion score 20 and above. The identified dimethyl‐labeled peptides are listed in Tables S2 and S3 (Supporting information). Finally, we tested three samples in each sample set, and only the peptides detected in all three samples are reported in this study.

### A similar pattern of proteins involved in disulfide bond formation is found in human and mouse cataract and *in vitro* modeling by oxidation with H_2_O_2_

**Figure 2:**
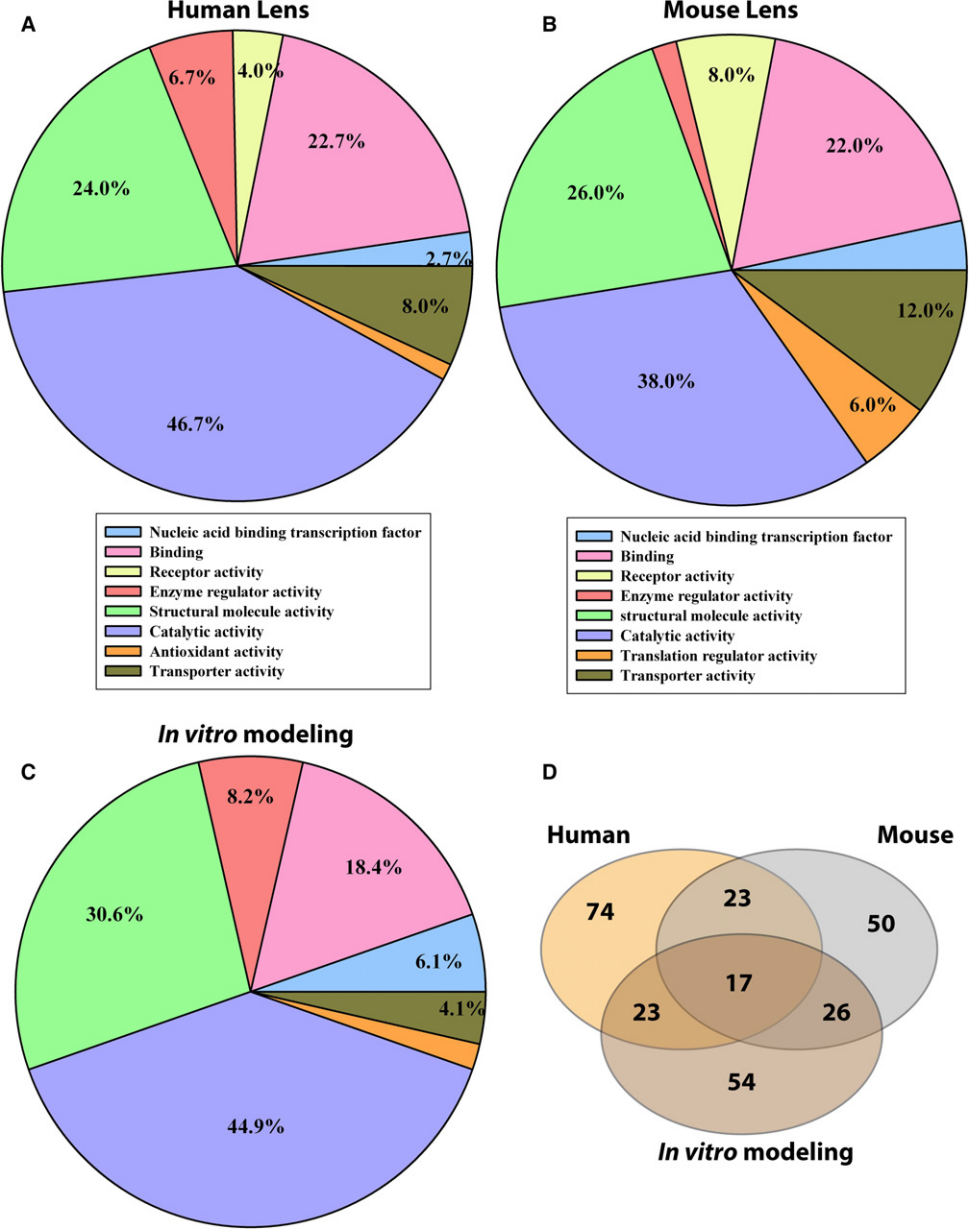
Protein categorization based on molecular functions and shared protein informatics between human, mouse, and *in vitro* model. The protein functional categorization was achieved with the Panther classification system (www.pantherdb.org). (A) Human lens protein functional pie graph. (B) LEGSKO/WT mouse lens protein functional pie graph. (C) Functional pie graph from *in vitro* oxidation of the mouse lens protein extract. (D) Shared protein identity between each sample set.

Seventy‐four oxidized proteins in either aged normal or aged cataractous human lenses (see Table 1), 50 proteins in either LEGSKO or WT mouse lenses (see Table 2), and 54 proteins in either nonoxidized or *in vitro*‐oxidized mouse lens protein extract (see Table S1, Supporting information) were detected by ICAT proteomic analysis. These were categorized based on their molecular functions using the Panther classification system (www.pantherdb.org). As illustrated in Fig. 2A–C, 46.7%, 38.0%, and 44.9% of disulfide‐forming proteins that were identified in human lens, mouse lens, and *in vitro* oxidation, respectively, are those with catalytic activity, such as the enzymes, glyceraldehyde‐3‐phosphate dehydrogenase (GAPDH), sorbitol dehydrogenase (SORD), and others. The second most prevalent group includes structural proteins, with 24.0%, 26.0%, and 30.6% of proteins identified in human, mouse lenses, and *in vitro* modeling experiments, respectively. These include several structural proteins unique to the lens, such as beaded filament structural protein 1 and 2 (BFSP1 and BFSP2). We also found a large number of binding proteins involved in disulfide bond formation, which accounted for 22.7%, 22.0%, and 18.4% in human, mouse, and *in vitro* modeling of the proteins, respectively. We also found proteins with enzyme regulator activity, nucleic acid binding transcription factor, receptor activity, transporter activity, transporter regulatory activity, and antioxidant activity‐related functions (see Fig. 2A–C, and Tables 1 and 2 and S1, Supporting information). Overall, of 50–74 proteins, 17 were found in all three sets of pools (see Table 3), and 23 proteins shared the same identity in human vs. mouse lens *in vivo,* while 23 proteins shared the same identity in human lens vs. *in vitro* data, and 26 proteins shared the same identity between mouse *in vivo* and *in vitro* modeling (Fig. 2D).

**Table 1:** Relative amount of disulfide bonding in peptides from proteins of aged normal and cataractous human lenses versus young lenses

Protein name	Description	Peptide Sequence^a^,^b^	ICAT_Aged_/ICAT_Young_ or ICAT_Cat_/ICAT_Young_ c,d,e,f,g				
			Aged Normal	Type II cataract	Type III cataract	Type IV cataract	Type V cataract
Antioxidant Activity							
PRDX6	Peroxiredoxin 6	DFTPVC47TTELGR	0.03 (± 0.003)	0.02 (± 0.001)	0.02 (± 0.001)	0.025 (± 0.001)	0.01 (± 0.001)
Calcium Binding							
DSG1	Desmoglein‐1	IHSDC77AANQQVTYR	0.64 (± 0.07)	3.02 (± 0.39)	5.88 (± 0.29)	18.29 (± 1.11)	34.28 (± 5.67)
S100A7L2	Protein S100‐A7‐Like 2	ENFPNFLSGC58EK	ND	ND	ND	12.23 (± 1.91)	25.51 (± 0.98)
S100A7A		QSHGAAPC96SGGSQ	0.45 (± 0.11)	0.66 (± 0.9)	0.89 (± 0.21)	0.55 (± 0.08)	0.67 (± 0.12)
		ENFPNFLSAC47DK	ND	0.69 (± 0.06)	1.04 (± 0.08)	1.89 (± 0.22)	2.22 (± 0.72)
S100AB		LLETEC66PQYIR	>1000	>1000	>1000	>1000	>1000
Catalytic Activity							
AMDHD1	Imidazolonepropionase‐related	C300SAILLPTTAYMLR	ND	18.90 (± 2.38)	41.12 (± 6.01)	85.4 (± 10.98)	>1000
CDA	Cytidine deaminase	SAYC31PYSHFPVGAALLTQEGR	ND	17.18 (± 2.21)	54.34 (± 9.11)	123.45(13.31)	112.35 (21.11)
FAH	Fumarylacetoacetase	IGFGQC408AGK	ND	2.62 (± 0.12)	5.70 (± 0.31)	6.02 (± 0.31)	8.05 (± 1.21)
		GEGMSQAATIC315K	0.002	0.0015	0.002	0.004	0.002
CDC42	Cell division control protein 42 homolog	YVEC157SALTQK	ND	ND	>1000	>1000	>1000
		C6VVVGDGAVGK	ND	ND	ND	20.74	39.11
RHOG	Pho‐related GTP‐binding protein Rhog	C6VVVGDGAVGK	ND	1.83 (± 0.31)	2.34 (± 0.73)	6.88 (± 1.11)	12.78 (± 1.37)
RAC1	Ras‐related C3 botulinum toxin substrate 1	YLEC157SALTQR	ND	ND	ND	ND	56.81818182
MFN2	Mitofusin 2	C188PLLK	ND	ND	ND	25.25 (± 5.62)	>1000
UCHL1	Ubiquitin C‐terminal hydrolase L1	FSAVALC220K	13.56 (± 1.19)	20.79 (± 1.02)	17.45 (± 4.15)	56.17 (± 8.98)	98.03 (± 9.72)
SERPINB6	Serpin peptidase inhibitor, clade B (ovalbumin), member 6	TNGILFC374GR	ND	5.77 (± 0.26)	12.43 (± 0.74)	20.04 (± 2.17)	71.94 (± 7.29)
		SC104DFLSSFR	ND	13.33 (± 0.97)	39.52 (± 1.89)	140.84 (± 27.47)	204.08 (± 18.91)
		NVFFSPMSMSCALAMVYMGAK	ND	ND	ND	93.45 (± 6.39)	>1000
SERPINB9	Serpin peptidase inhibitor, clade B (ovalbumin), member 9	ANSILFC370GR	6.28 (± 0.34)	20.04 (± 4.27)	19.72 (± 3.12)	43.29 (± 7.98)	71.94 (± 5.39)
ABCA10	ATP binding cassette subfamily A member 10	C679SDQGIR	ND	ND	ND	121.9512195	>1000
TPI1	Triosephosphate isomerase 1	IIYGGSVTGATC218K	ND	23.41 (± 1.04)	29.78 (± 2.29)	47.32 (± 9.81)	70.92 (± 7.74)
LGSN	Lengsin, lens protein with glutamine synthetase domain	VIC170DTFTVTGEPLLTSPR	ND	ND	22.83 (± 0.81)	129.8 (± 4.19)	>1000
GSS	Glutathione synthetase	C294PDIATQLAGTK	2.17 (± 0.36)	77.57 (± 11.38)	>1000	>1000	>1000
ENO1	Enolase 1	FGANAILGVSLAVC26K	0.12 (±0.02)	0.78 (± 0.06)	1.04 (± 0.02)	1.12 (± 0.27)	1.33 (± 0.19)
		TGAPC306R	0.011	0.013	0.019	0.011	0.89 (± 0.02)
SORD	Sorbitol dehydrogenase	VLVC179GAGPIGMVTLLVAK	ND	109 (± 7.37)	>1000	>1000	>1000
		HNAAFC140YK	ND	322.58 (± 21.88)	>1000	>1000	>1000
		LPDNVTFEEGALIEPLSVGIHAC165R	ND	ND	ND	>1000	>1000
ALDH1A1	Aldehyde dehydrogenase 1 family member A1	IGPALSC186GNTVVVKPAEQTPLTALHVASLIK	ND	3.9 (± 0.98)	22.02 (± 3.29)	13.71 (± 7.72)	15.33 (± 2.78)
		LYSNAYLNDLAGC126IK	ND	45.45 (± 6.48)	70.92 (± 14.26)	44.64 (± 21.58)	75.75 (± 8.90)
GMPR	Guanosine monophosphate reductase	STC316TYVGAAK	ND	3.16 (± 0.33)	5.54 (± 0.72)	6.18 (± 1.17)	15.62 (± 4.01)
CBR1	Carbonyl reductase 1	SC150SPELQQK	39.37 (± 2.21)	69.44 (± 11.16)	41.15 (± 8.89)	64.93 (± 13.37)	23.14 (± 5.53)
GAPDH	Glyceraldehyde‐3‐phosphate dehydrogenase	VPTANVSVVDLTC247R	ND	23.25 (± 0.78)	44.24 (± 3.46)	73.52 (± 8.02)	>1000
HPD	4‐hydroxyphenylpyruvate dioxygenase	QAASFYC37SK	2.48 (± 0.17)	18.34 (± 1.74)	13.47 (± 2.87)	11.33 (± 2.44)	65.35 (± 8.83)
LDHA	Lactate dehydrogenase	VIGSGC105NLDSAR	ND	ND	181.81 (± 23.26)	112.35 (± 17.27)	>1000
MDH1	Malate dehydrogenase 1	AIC269DHVR	>1000	>1000	>1000	>1000	>1000
PRDX6	Peroxiredoxin 6	DFTPVC47TTELGR	0.01 (± 0.003)	0.02 (± 0.001)	0.03 (± 0.001)	0.025 (± 0.001)	0.02 (± 0.001)
TKT	Transketolase	TVPFC386STFAAFFTR	ND	476.19 (± 19.78)	>1000	>1000	>1000
AKR1B1	Aldo‐keto reductase family 1, member B1 (aldose reductase)	LIQYC200QSK	ND	ND	ND	ND	312.5 (± 22.43)
NME1	NME/NM23 nucleoside diphosphate kinase 1	GDFC109IQVGR	ND	ND	ND	ND	16.55 (± 1.33)
PGK1	Phosphoglycerate kinase 1	AC108ANPAAGSVILLENLR	ND	131.57 (± 9.72)	>1000	>1000	>1000
PCMT1	Protein‐L‐isoaspartate (D‐aspartate) O‐methyltransferase	ALDVGSGSGILTAC153FAR	ND	102.04 (± 11.21)	>1000	>1000	>1000
		MVGC160TGK	ND	ND	ND	ND	200 (± 17.71)
IPO5	Importin 5	TIEC578ISLIGLAVGK	ND	22.37 (± 2.22)	>1000	>1000	>1000
PARK7	Parkinsonism‐associated deglycase	DPVQC46SR	4.87 (± 0.78)	30.48 (± 2.28)	34.60 (± 4.21)	70.92 (± 7.84)	>1000
		DVVIC53PDASLEDAKK	ND	>1000	>1000	>1000	>1000
RNF149	Ring finger protein 149	GGC118TFKDK	ND	ND	ND	ND	434.13 (± 24.89)
Enzyme regulator protein							
PLCG1	Phospholipase C gamma 1	SSLRGLEPC1088AISIEVLGAR	ND	38.75 (± 4.42)	41.15 (± 2.87)	48.78 (± 7.73)	64.93 (± 5.41)
Nucleic acid binding transcription factor (PARK7 can also be categorized in this section)							
WIZ	Widely interspaced zinc finger motifs	C1456VFGTNSSRAYVQHAKLHMR	ND	ND	13.19 (± 1.02)	>1000	>1000
Receptor Activity							
LSAMP	Limbic system‐associated membrane protein	VDVYDEGSYTC111SVQTQHEPK	0.07	0.04	0.14	0.27	0.98 (± 0.03)
		C53VVEDK	0.33	0.23	0.42	0.41	1.13 (± 0.06)
PLA2R1	Phospholipase A2 receptor 1	C51IQAGK	1.35 (± 0.04)	1.77 (± 0.26)	7.56 (± 1.17)	21.42 (± 7.52)	27.08 (± 9.13)
COL4A1	Collagen type IV alpha 1	SAPFIEC1616HGR	0.24	8.13 (± 1.12)	34.28 (± 5.24)	63.79 (± 13.21)	>1000
Structure (COL4A1 can also be categorized in this section)							
FBN1	Fibrillin 1	C1171VNLIGK	37.91 (± 5.17)	234.67 (± 30.14)	>1000	>1000	>1000
KRT6A	Keratin 6A	GSGGLGGAC51GGAGFGSR	1.23 (± 0.21)	7.86 (± 0.93)	12.56 (± 1.73)	42.55 (± 9.21)	>1000
		ISIGGGSC77AISGGYGSR	0.68 (± 0.01)	1.32 (± 0.32)	15.32 (± 2.19)	8.91 (± 1.21)	50.5 (± 8.74)
		LLEGEEC474R	ND	2.26 (± 0.31)	31.45 (± 6.30)	247.61 (± 30.13)	>1000
BFSP1	Beaded filament structural protein 1	SSYDC167R	5.9 (± 0.13)	46.94 (± 2.05)	39.21 (± 1.74)	57.47 (± 4.03)	101.01 (± 8.02)
ACTG1/ACTB	Actin gamma 1/actin, beta	C285DVDIR	4.02 (± 0.47)	24.81 (± 2.33)	53.47 (± 3.92)	47.28 (± 11.43)	119.04 (± 14.01)
		LC217YVALDFEQEMATAASSSSLEK	ND	ND	21.27 (± 1.11)	32.01 (± 6.02)	39.19 (± 4.91)
ACTBL2	Actin, beta‐like 2	C286DVDIR	4.02 (± 0.47)	24.81 (± 2.33)	53.47 (± 3.92)	47.28 (± 11.43)	119.04 (± 14.01)
KRT2	Keratin 2	STSSFSC42LSR	0.83 (± 0.05)	3.40 (± 0.43)	14.90 (± 3.01)	102.48 (± 9.02)	>1000
		LLEGEECR	ND	2.26 (± 0.11)	52.37 (± 3.57)	531.92 (± 48.23)	>1000
KRT10	Keratin 10	SGGGGGGGGC25GGGGGVSSLR	0.30	0.17	1.03 (± 0.08)	3.56 (± 0.13)	8.78 (± 1.03)
		GSSGGGC66FGGSSGGYGGLGGFGGGSFR	0.49	2.29 (± 0.10)	4.05 (± 0.47)	30.95 (± 2.03)	30.30 (± 1.79)
		AETEC427QNTEYQQLLDIK	ND	2.27	7.71 (± 1.03)	16.12 (± 3.25)	18.19 (± 6.03)
KRT5	Keratin 5	VSLAGAC55GVGGYGSR	6.82 (± 0.84)	3.48 (± 0.99)	10.03 (± 1.27)	29.58 (± 1.71)	96.15 (± 10.03)
		LLEGEEC474R	ND	2.26(±0.72)	37.04 (± 7.99)	428.11 (± 21.05)	>1000
KRT9	Keratin 9	QEIEC432QNQEYSLLLSIK	ND	2.84 (± 0.81)	2.02 (± 0.33)	2.58 (± 0.49)	5.17 (± 0.81)
KRT14	Keratin 14	ISSVLAGGSC40R	4.22 (± (0.72)	1.71 (± 0.93)	6.13 (± 2.13)	24.57 (± 4.01)	32.36 (± 2.11)
		GSC18GIGGGIGGGSSR	3.37 (± 0.21)	5.51 (± 0.78)	7.92 (± 1.03)	19.84 (± 3.41)	>1000
		C389EMEQQNQEYK	3.98 (± 0.45)	2.01 (± 0.19)	14.90 (± 2.05)	120.48 (± 13.48)	>1000
KRT1	Keratin 1	FSSC49GGGGGSFGAGGGFGSR	2.16 (± 0.77)	3.38 (± 0.69)	5.74 (± 1.20)	27.17 (± 2.31)	14.34 (± 2.09)
		MSGEC497APNVSVSVSTSHTTISGGGSR	3.64 (± 0.91)	2.17 (± 0.67)	3.78 (± 1.28)	12.95 (± 2.10)	21.33 (± 3.00)
BFSP2	Beaded filament structural protein 2, phakinin	VELHNTSC326QVQSLQAETESLR	4.26 (± 0.47)	4.99 (± 0.23)	7.32 (± 1.73)	13.77 (± 2.21)	42.13 (± 7.99)
		APGVYVGTAPSGC65IGGLGAR	ND	ND	21.00 (± 4.02)	77.13 (± 10.10)	>1000
PLEC	Plectin	LQLEAC1026ETR	ND	>1000	>1000	>1000	>1000
SPTAN1	Spectrin alpha, nonerythrocytic 1	ALC315AEADR	ND	ND	ND	ND	64.51 (± 4.21)
DSP	Desmoplakin	NQC1206TQVVQER	0.53 (± 0.02)	2.57 (± 0.13)	14.78 (± 2.37)	372.5 (± 21.02)	>1000
		YQAEC1069SQFK	ND	2.43 (± 0.08)	7.48 (± 0.92)	31.01 (± 2.09)	>1000
		QMGQPC135DAYQK	ND	2.70 (± 0.10)	4.11 (± 0.27))	3.72 (± 0.92)	>1000
PRC1	Protein regulator of cytokinesis 1	QLEMQKSQNEAVC195EGLR	ND	ND	ND	ND	38.45 (± 3.12)
LIM2	Lens intrinsic membrane protein 2	YC48LGNK	0.28	0.75 (± 0.01)	0.62 (± 0.03)	0.73 (± 0.02)	0.20 (± 0.01)
Transporter activity (ABCA10, AKR1B1, IPO5, LIM2 and COL4A1 can also be categorized in this section)							
SCNN1B	Sodium channel epithelial 1 beta subunit	IIC43EGPKKK	ND	>1000	>1000	>1000	>1000
Protein ubiquitination							
RNF149	Ring finger protein 149	GGC118TFKDK	ND	ND	ND	ND	434.13 (± 24.89)
Other function							
FABP5	Fatty acid binding protein 5	TTQFSC67TLGEK	12.21 (± 0.17)	15.24 (± 0.09)	27.88 (± 1.05)	38.75 (± 1.37)	47.84 (± 4.92)
ALDOA	Aldolase, fructose‐bisphosphate A	RC202QYVTEK	7.71 (± 1.12)	27.47 (± 3.26)	32.57 (± 2.87)	54.43 (± 8.01)	48.54 (± 7.23)
		ALANSLAC339QGK	3.99 (± 0.23)	29.67 (± 0.72)	>1000	>1000	>1000
		YASIC178QQNGIVPIVEPEILPDGDHDLK	ND	ND	ND	>1000	>1000
MIF	Macrophage migration inhibitory factor	LLC81GLLAER	4.34 (± 0.11)	16.26 (± 0.41)	37.18 (± 2.08)	66.66 (± 9.77)	75.75 (± 10.32)
ALB	Albumin	YIC289ENQDSISSK	0.04	0.15	0.76	0.92 (± 0.03)	6.79 (± 0.37)
		RPC511FSALEVDETYVPK	ND	ND	ND	ND	>1000
BCORL1	\BCL6 corepressor‐like 1	C1121GKEK	ND	ND	ND	ND	9.11 (± 0.53)
FNDC3A	Fibronectin type III domain containing 3A	C211PSPINEHNGLIK	1.03	1.05 (± 0.02)	1.01 (± 0.01)	1.92 (± 0.22)	4.21 (± 0.37)
PRDM1	PR domain 1	NELC239PK	ND	ND	ND	ND	>1000
YWHAZ	Tyrosine 3‐monooxygenase	DIC94NDVLSLLEK	ND	6.94 (± 0.74)	44.89 (± 2.38)	>1000	>1000
RBP1	Retinol binding protein 1	VEGVVC189K	ND	>1000	>1000	>1000	>1000
PKM	Pyruvate kinase	GIFPVLC548K	2.8 (± 0.03)	12.2 (± 0.28)	54.94 (± 7.12)	91.34 (± 10.89)	77.1 (± 13.54)
		NTGIIC123TIGPASR	ND	>1000	>1000	>1000	>1000
		AGKPVIC400ATQMLESMIK	ND	ND	ND	>1000	>1000
HBB	Hemoglobin subunit beta	GTFATLSELHC94DK	ND	ND	ND	43.66 (± 3.12)	188.67 (± 5.01)
MFAP2	Microfibrillar‐associated protein 2	EEQYPC103TR	0.32	0.21	0.42	0.35	1.27 (± 0.02)
PEBP1	Phosphatidylethanolamine binding protein 1	C133DEPILSNR	0.14	0.42 (± 0.01)	0.77 (± 0.02)	3.22 (± 0.13)	7.18 (± 0.42)
SPTBN1	Spectrin beta, nonerythrocytic 1	IHC112LENVDK	ND	1.26 (± 0.11)	45.79 (± 8.91)	87.18 (± 10.06)	>1000
		NDSFTTC1970IELGK	ND	ND	16.41 (± 5.03)	55.23 (± 3.07)	>1000

a The amino acid sequence number of the cysteine residues was assigned by counting the N‐terminal methionine as residue 1.

b The peptides from crystallins were excluded.

c This ratio of disulfides in normal aged and cataractous lens vs. young normal control was determined as ICAT‐^13^C/ICAT‐^12^C.

d The ICAT ratio has been adjusted based on dimethyl labeling results.

e The number in parenthesis is the value of standard error (SE).

f ND means not detectable.

g Value <1 indicates the less disulfide bond formation in aged normal and aged cataractous lenses compared to control; value>1000 indicates the significant abundant peak identified in MS is by heavy ICAT label.

**Table 2:** Relative amount of disulfide bonding in peptides from proteins of LEGSKO mouse lenses vs. age‐matched control lenses

Protein name	Description	Sequence^a^,^b^	ICAT_LEGSKO_/ICAT_WT_ c,d,e,f	Homologous motif found in human
Binding				
FBLN1	Fibulin 1	DC50SLPYTSESK	64.10 (± 8.18)	
		LGESC234INTVGSFR	25.06 (± 4.69)	
PARK7	Parkinsonism‐associated deglycase	DPVQC46SR	13.93 (± 2.17)	Cys46
EIF4A1	Eukaryotic translation initiation factor 4A1	AILPC66IK	14.95 (± 1.82)	
SPTAN1	Spectrin alpha, nonerythrocytic 1	GAC1627AGSEDAVK	68.51 (± 11.03)	
RAC1	Ras‐related C3 botulinum toxin substrate 1	YLEC157SALTQR	14.26 (± 3.17)	Cys157
IPO5	Importin 5	TIEC560ISLIGLAVGK	>1000	Cys578
EEF2	Eukaryotic translation elongation factor 2	YVEPIEDVPC466GNIVGLVGVDQFLVK	11.96 (± 1.15)	
		STLTDSLVC41K	4.78 (± 0.78)	
		TFC290QLILDPIFK	18.80 (± 3.55)	
CLU	Clusterin	EGEDDRTVC284K	431.95 (± 37.01)	
MLXIP	MLX interacting protein	TSSC118HLSIDASLTK	521.21 (± 21.19)	
FBN1	Fibrillin 1	C1173VNLIGK	>1000	
		NGEC2420VNDR	625.00 (± 70.36)	
		C2232PVGYVLR	>1000	
		C1960NEGYEVAPDGR	6.24 (± 0.44)	
LTBP2	Latent transforming growth factor beta binding protein 2	VVFTPTIC365K	>1000	
Catalytic activity (PARK7, EIF4A1, RAC1, IPO5, and EEF2 can also be categorized in this section)				
CTSB	Cathepsin B	SC211EAGYSPSYK	34.25 (± 3.01)	
TPI1	Triosephosphate isomerase 1	IAVAAQNC117YK	>1000	
		IIYGGSVTGATC268K	12.38 (± 2.22)	Cys218
GSS	Glutathione synthetase	C225PDIAIQLAGTK	>1000	Cys294
HTRA1	HtrA serine peptidase 1	TYTNLC130QLR	5.30 (± 1.17)	
ENO3	Enolase 3	TGAPC399R	7.93 (± 0.31)	
		VNQIGSVTESIQAC357K	6.47 (± 1.00)	
TKT	Transketolase	TVPFC386STFAAFFTR	6.76 (± 0.83)	Cys386
GAPDH	Glyceraldehyde‐3‐phosphate dehydrogenase	AAIC48SGK	40.03 (± 7.38)	
		VPTPNVSVVDLTC271R	13.67 (± 3.11)	Cys247
HACE1	HECT domain and ankyrin repeat containing E3 ubiquitin protein ligase 1	GANPNYQDISGC99TPLHLAAR	3.09 (± 0.12)	
PHGDH	Phosphoglycerate dehydrogenase	VVNC234AR	9.43 (± 0.88)	
		ALVDHENVISC281PHLGASTK	3.11 (± 0.15)	
		ALQSGQC254AGAALDVFTEEPPRDR	4.3 (± 0.50)	
ASS1	Argininosuccinate synthase 1	FELTC132YSLAPQIK	9.92 (± 2.06)	
		YLLGTSLARPC97IAR	17.51 (± 9.91)	
LGSN	Lengsin	TNMFC383SGSGVER	62.72 (± 7.25)	
		VIC224DTFTVTGEPLLTSPR	8.27 (± 1.11)	Cys170
		DLKDSVPTTWGYNDNSC445ALNIK	3.08 (± 0.39)	
PAICS	Phosphoribosylaminoimidazole carboxylase	ITSC63IFQLLQEAGIK	5.38 (± 0.20)	
ALDH1A1	Aldehyde dehydrogenase 1 family member A1	IGPALSC187GNTVVVKPAEQTPLTALHLASLIK	21.94 (± 1.17)	Cys186
		VFANAYLSDLGGC126IK	22.36 (± 7.33)	Cys126
		LEC370GGGR	22.83 (± 3.06)	
UCHL1	Ubiquitin C‐terminal hydrolase L1	FSAVALC220K	12.29 (± 1.04)	Cys220
		NEAIQAAHDSVAQEGQC152R	4.18 (± 0.37)	Cys152
Enzyme regulator activity (IPO5 and EEF2 can also be categorized in this section)				
Nucleic acid binding transcription factor activity (PARK7 and MIXIP)				
Receptor activity (COL4A1 can also be categorized in this section)				
COL4A2	Collagen, type IV, alpha 2	GTC1660HYFANK	146.48 (± 9.73)	
		ATPFIEC1653NGGR	>1000	
		AHNQDLGLAGSC1532LAR	362.33 (± 47.11)	
		HSQTDQEPMC1499PVGMNK	635.14 (± 104.12)	
HSPG2	Heparan sulfate proteoglycan 2	AHSVEEC731R	56.1 (± 9.14)	
		LPAIEPSDQGQYLC1932R	>1000	
		SQSVRPGADVTFIC1792TAK	454.55 (± 21.37)	
COL4A4		AAPFVEC1626QGR	12.95 (± 0.28)	
Structure molecular activity (Col4a1, Sptan1, Col4a2, Fbn1, and Ltbp2 can also be categorized in this section)				
VIM	Vimentin	QVQSLTC328EVDALK	26.2 (± 3.41)	
KRT14	Keratin 14	C334EMEQQNQEYK	1.88 (± 0.10)	Cys389
COL4A2	Collagen, type IV, alpha 2	GTC1660HYFANK	146.48 (± 11.04)	
		ATPFIEC1653NGGR	>1000	
		AHNQDLGLAGSC1532LAR	362.33 (± 34.04)	
		HSQTDQEPMC1499PVGMNK	635.14 (± 37.92)	
ACTBL2	Actin, beta‐like 2	C285DVDIR	14.7 (± 2.03)	Cys285
COL4A4	Collagen type IV alpha 4	AAPFVEC1626QGR	12.95 (± 0.93)	
TUBA1A	Tubulin alpha 1a	TIQFVDWC347PTGFK	1.78 (± 0.08)	
ACTB	Actin, beta	C285DVDIR	14.7 (± 0.62)	Cys285
		LC217YVALDFEQEMATAASSSSLEK	8.92 (± 2.07)	
LIM2	Lens intrinsic membrane protein 2	YC46LGNK	1.83 (± 0.12)	Cys48
BFSP1	Beaded filament structural protein 1	SSYDC290R	8.48 (± 1.14)	Cys167
Translation regulator activity (EIF4A1 and EEF2 can also be categorized in this section)				
Transporter activity (COL4A1, IPO5, COL4A2, COL4A4, and LIM2 can also be categorized in this section)				
ATP1B3	ATPase Na^+^/K^+^ transporting subunit beta 3	IIDLIPDGYPQISC191LPK	4.66 (± 0.46)	
Other functions				
FABP5	Fatty acid binding protein 5	TTVFSC67NLGEK	6.72 (± 0.72)	Cys67
		TETVC87TFQDGALVQHQQWDGK	6.23 (± 0.25)	
MIF	Macrophage migration inhibitory factor	LLC81GLLAER	31.17 (± 2.14)	Cys81
ALB	Albumin	Raelakymc289e nqatissk	88.13 (± 7.13)	Cys289
		rp c511fsaltvdet yvpk	167.03 (± 9.73)	Cys511
		LC99AIPNLR	88.49 (± 5.29)	
		TNC416DLYEK	105.26 (± 15.32)	
		DTC591FSTEGPNLVTR	22.46 (± 3.61)	
		VC485LLHEK	>1000	
PKM	Pyruvate kinase	GIFPVLC474K	13.86 (± 0.88)	Cys548
		NTGIIC49TIGPASR	19.76 (± 2.04)	Cys123
		AGKPVIC326ATQMLESMIK	2.64 (± 0.09)	
MFAP2	Microfibrillar‐associated protein 2	TVC144AHEELLR	125.00 (± 8.32)	
PFN1	Profilin 1	C128YEMASHLR	45.73 (± 3.83)	
VTN	Vitronectin	INC214QGK	233.23 (± 17.17)	
		C28TQGFMASK	4.88 (± 0.67)	
		GQYC179YELDETAVRPGYPK	6.68 (± 0.33)	
NID1	Nidogen 1	C837MPGEVSK	25.70 (± 1.37)	
		AEC1133LNPAQPGR	140.84 (± 5.82)	
		QC423VAEGSPQR	5.16 (± 0.92)	
NDUFA11	NADH dehydrogenase	EKPDDPLNYFIGGC97AGGLTLGAR	36.90 (± 2.18)	
CCT4	Chaperonin containing TCP1 subunit 4	IGLIQFC252LSAPK	9.53 (± 1.03)	
IDH1	Isocitrate dehydrogenase 1	C73ATITPDEK	0.009 (± 0.001)	
HSP90AB1	Heat‐shock protein 90 kDa alpha family class B member 1	FENLC564K	106.38 (± 13.06)	

a The amino acid sequence number of the cysteine residues was assigned by counting the N‐terminal methionine as residue 1.

b The peptides from crystallins were excluded.

c This ratio of disulfides in 9‐month‐old LEGSKO and age‐matched wild‐type whole lenses was determined as ICAT‐^13^C/ICAT‐^12^C.

d The ICAT ratio has been adjusted based on dimethyl labeling results.

e The number in parenthesis is the value of standard error (SE).

f Value <1 indicates the less disulfide bond formation in aged normal and aged cataractous lenses compared to control; value>1000 indicates the significant abundant peak identified in MS is by heavy ICAT label.

**Table 3:** Summary of disulfide‐forming proteins and peptides common to human, mouse, and *in vitro* oxidation

Protein Name	Description	Human peptide sequence	LEGSKO mouse peptide sequence	Mouse *in vitro* peptide sequence
KRT6A	Keratin 6A	GSGGLGGAC51GGAGFGSR	–	–
		ISIGGGSC77AISGGYGSR	–	–
		LLEGEEC474R	–	LLEGEEC463R
BFSP1	Beaded filament structural protein 1	SSYDC167R	SSYDC290R	SSYDC290R
		–	–	RPC573PVIIPGPDEPSTSHSQTSGSNQGGPVGPASK
SPTAN1	Spectrin alpha, nonerythrocytic 1	ALC315AEADR	–	–
		–	GAC1627AGSEDAVK	GAC1627AGSEDAVK
AKR1B1	Aldo‐keto reductase family 1, member B1 (aldose reductase)	LIQYC200QSK	–	–
		–	–	HIDC45AQVYQNEK
LIM2	Lens intrinsic membrane protein 2	YC48LGNK	YC46LGNK	YC46LGNK
UCHL1	Ubiquitin C‐terminal hydrolase L1	FSAVALC220K	FSAVALC220K	FSAVALC220K
		–	NEAIQAAHDSVAQEGQC152R	NEAIQAAHDSVAQEGQC152R
TPI1	Triosephosphate isomerase 1	IIYGGSVTGATC218K	IIYGGSVTGATC268K	–
			IAVAAQNC117YK	IAVAAQNC117YK
KRT1	Keratin 1	FSSC49GGGGGSFGAGGGFGSR	–	–
		MSGEC497APNVSVSVSTSHTTISGGGSR	–	–
		–	C334EMEQQNQEYK	C334EMEQQNQEYK
MFAP2	Microfibrillar‐associated protein 2	EEQYPC103TR	–	–
		–	TVC144AHEELLR	–
ACTG1/ACTB	Actin gamma 1	C285DVDIR	C285DVDIR	C163DVDIR
		LC217YVALDFEQEMATAASSSSLEK	LC217YVALDFEQEMATAASSSSLEK	C285DVDIR
RAC1	Ras‐related C3 botulinum toxin substrate 1	YLEC157SALTQR	YLEC157SALTQR	LC217YVALDFEQEMATAASSSSLEK
LGSN	Lengsin, lens protein with glutamine synthetase domain	VIC170DTFTVTGEPLLTSPR	VIC224DTFTVTGEPLLTSPR	VIC224DTFTVTGEPLLTSPR
		–	TNMFC383SGSGVER	TNMFC383SGSGVER
		–	DLKDSVPTTWGYNDNSC445ALNIK	DLKDSVPTTWGYNDNSC445ALNIK
		–	–	ATC196FNSDIVLMPELSTFR
ACTBL2	Actin, beta‐like 2	C286DVDIR	C286DVDIR	C286DVDIR
PKM	Pyruvate kinase	GIFPVLC548K	GIFPVLC474K	GIFPVLC474K
		NTGIIC123TIGPASR	NTGIIC49TIGPASR	NTGIIC49TIGPASR
		AGKPVIC400ATQMLESMIK	AGKPVIC326ATQMLESMIK	AGKPVIC326ATQMLESMIK
		–	–	AEGSDVANAVLDGADC358IMLSGETAKGDYPLEAVR
PPIA	Peptidylprolyl isomerase A	GSC52FHR	–	–
		–		IIPGFMC62QGGDFTR
FBN1	Fibrillin 1	C1171VNLIGK	C1173VNLIGK	–
		–	NGEC2420VNDR	–
		–	C2232PVGYVLR	–
		–	C1960NEGYEVAPDGR	–
ALDH1A1	Aldehyde dehydrogenase 1 family member A1	IGPALSC186GNTVVVKPAEQTPLTALHVASLIK	IGPALSC187GNTVVVKPAEQTPLTALHLASLIK	IGPALSC187GNTVVVKPAEQTPLTALHLASLIK
		LYSNAYLNDLAGC126IK	VFANAYLSDLGGC126IK	VFANAYLSDLGGC126IK
		–	LEC370GGGR	–
ENO1	Enolase 1	FGANAILGVSLAVC26K	VNQIGSVTESLQAC357K	–
		TGAPC306R	–	–
KRT5	Keratin 5	VSLAGAC55GVGGYGSR	–	–
		LLEGEEC474R	LLEGEEC473R	–
TKT	Transketolase	TVPFC386STFAAFFTR	TVPFC386STFAAFFTR	–
IPO5	Importin 5	TIEC578ISLIGLAVGK	TIEC560ISLIGLAVGK	–
FABP5	Fatty acid binding protein 5	TTQFSC67TLGEK	TTVFSC67NLGEK	TTVFSC67NLGEK
		–	TETVC87TFQDGALVQHQQWDGK	TETVC87TFQDGALVQHQQWDGK
GAPDH	Glyceraldehyde‐3‐phosphate dehydrogenase	VPTANVSVVDLTC205R	VPTPNVSVVDLTC271R	VPTPNVSVVDLTC271R
		–	AAIC48SGK	AAIC48SGK
PARK7	Parkinsonism‐associated deglycase	DPVQC46SR	DPVQC46SR	DPVQC46SR
		DVVIC53PDASLEDAKK	–	–
COL4A1	Collagen type IV alpha 1	SAPFIEC1616HGR	SAPFIEC1616HGR	–
		–	AHGQDLGTAGSC1493LR	–
GSS	Glutathione synthetase	C294PDIATQLAGTK	C225PDIAIQLAGTK	–
ALB	Albumin	YIC289ENQDSISSK	Raelakymc289e nqatissk	raelakymc289e nqatissk
		RPC511FSALEVDETYVPK	rp c511fsaltvdet yvpk	rp c511fsaltvdet yvpk
		–	LC99AIPNLR	LC99AIPNLR
		–	TNC416DLYEK	TNC416DLYEK
		–	DTC591FSTEGPNLVTR	DTC591FSTEGPNLVTR
		–	VC485LLHEK	VC485LLHEK
		–	–	AETFTFHSDIC538TLPEKEK
MIF	Macrophage migration inhibitory factor	LLC81GLLAER	LLC81GLLAER	–
ALDOA	Aldolase, fructose‐bisphosphate A	RC202QYVTEK	–	–
		ALANSLAC339QGK	–	ALANSLAC393QGK
		YASIC178QQNGIVPIVEPEILPDGDHDLK	–	YASIC232QQNGIVPIVEPEILPDGDHDLKR

### Significant cysteine residue oxidation occurs in lens intermediate filament and other structural proteins

**Figure 3:**
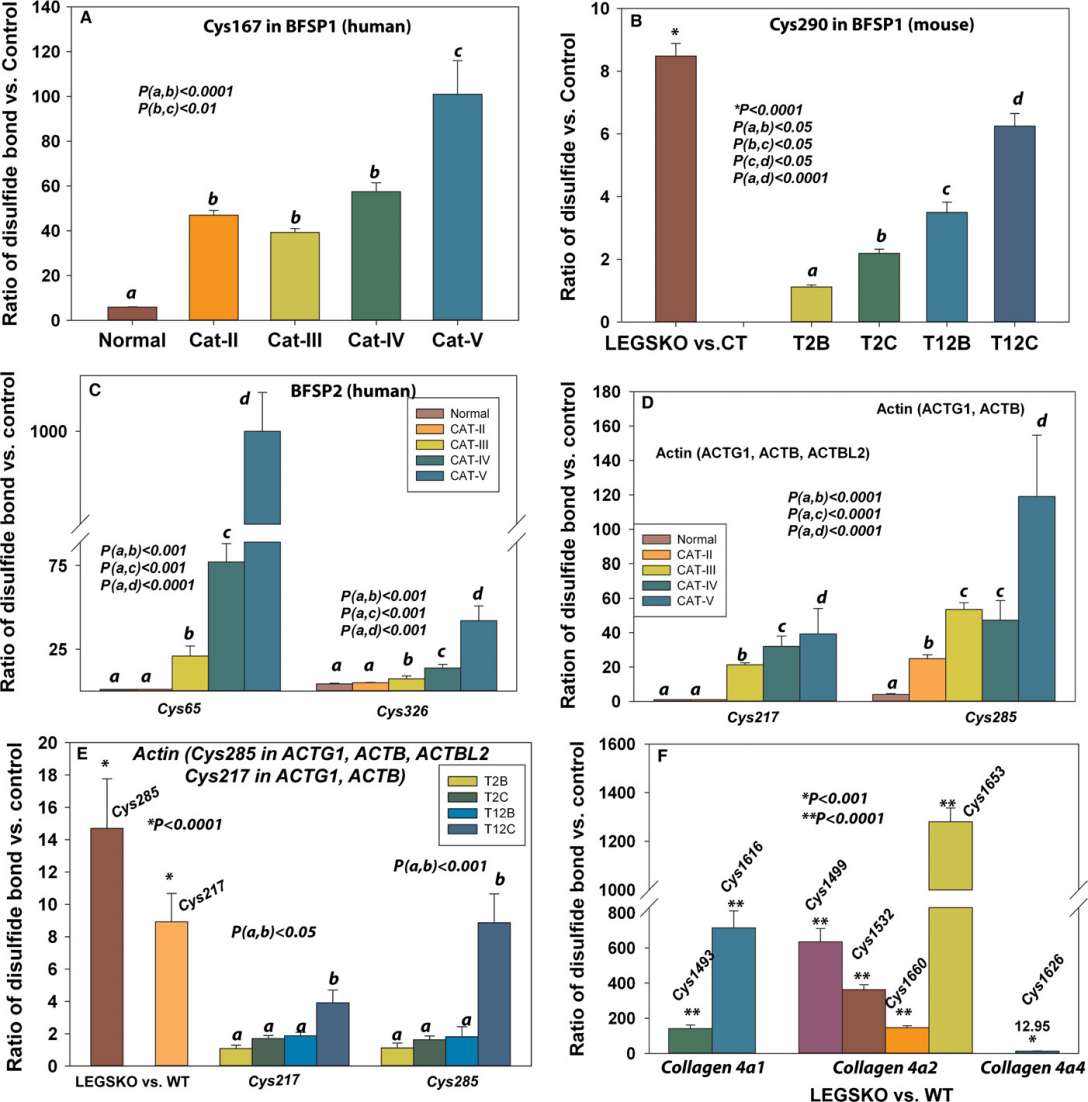
Representative proteins and their cysteine residues that are involved in disulfide cross‐linking in lens structural proteins. (A) The disulfide bond ratio of the Cys167 in filensin (BFSP1) in human lens. (B) The disulfide bond ratio of the Cys290 in mouse BFSP1 in both LEGSKO mouse and *in vitro* oxidation by H_2_O_2_ compared to their control. (C) The disulfide bond ratio of the Cys65 in human phakinin (BFSP2). (D) The Cys217 in various actins (ACTB, ACTG1, and ACTBL2) and the Cys285 in various actins (ACTB and ACTG1) in aged normal and cataractous human lenses compared to young normal control. (E) The actin cysteine residue Cys285 and Cys217 in both LEGSKO mouse and *in vitro* oxidation by H_2_O_2_ compared to their control. (F) Cys1493 and Cys1616 in collagen 4a1, Cys1419, Cys1532, Cys1660, and Cys1653 in collagen 4a2, and Cys1626 in collagen 4a4 were oxidized in LEGSKO vs. age‐matched WT mice lenses. Cat‐II: grade II cataract; Cat‐II: grade III cataract; Cat‐IV: grade IV cataract; Cat‐V: grade V cataract. T2B, T2C, T12B, and T12C are *in vitro* oxidation sample pairs (see Fig. 1). All ICAT data are expressed as the ratio vs. young lenses pool, LEGSKO vs. age‐matched WT, or oxidized vs. nonoxidized lens protein extract. Standard errors were calculated from three biological replicates. One‐way ANOVA was used to compare the significance between groups, and *P* < 0.05 was considered significant. The significance level was marked by either “*, **“ or lower case letters and the significance value was shown inside the figures.

Intermediate filaments (IFs) are playing a critical role in cell cytoskeleton integrity (Song *et al*., 2009). The cysteine residue 167 located in the filensin (BFSP1) central rod domain was oxidized into disulfide at moderate levels in aged human lenses, while significantly oxidized in category II to V cataractous lenses (Fig. 3A). The mouse filensin Cys290 with the same motif as Cys167 in the human was also found to be eightfold oxidized compared to age‐matched WT lenses (Fig. 3B). Interestingly, this was also reproduced by *in vitro* oxidation with H_2_O_2_ (Fig. 3B; also see Table S1, Supporting information). Similarly, the Cys65 located in the head domain of phakinin (BFSP2) was not detectable in aged normal and grade II cataractous human lenses, while intensively oxidized at grade III to V cataractous human lenses (Fig. 3C). The oxidation and disulfide formation at Cys326 located at the rod domain of phakinin was mild in old normal and category II cataractous human lenses but significantly oxidized in higher‐grade cataractous lenses (Fig. 3C). However, we did not detect phakinin disulfide cross‐linking from mouse and *in vitro* modeling samples. We also detected a large number of cysteine residues forming disulfide bond in cytokeratin proteins, particular in human lenses. This included Cys51, Cys77, and Cys474 in keratin 6A; Cys42 and Cys489 in keratin 2; Cys25, Cys66, and Cys427 in keratin 10; Cys55 and Cys474 in keratin 5; Cys432 in keratin 9; Cys40, Cys18, and Cys389 in keratin 14; and Cys49 and Cys497 in keratin 1 (Table 1). The majority of these cysteine residue oxidation sites were positively associated with degrees of cataract severity. Some of the cysteine residues in keratin 2 and keratin 14 were completely oxidized in grade V cataractous lenses (ratio >1000; Table 1). Although keratin oxidation, especially cysteine oxidation, has not been reported in the lens, several studies have implicated disulfide bonds in regulating the cytoskeletal assembly and organization in skin keratinocytes (Feng & Coulombe, 2015). This includes Cys40 in the head domain of keratin 14, which we also detected in the lens proteins.

Several IF‐associated structural proteins were also identified in our study, including lengsin, vimentin, and plectin. The oxidation of Cys1026 in plectin was not detectable in old normal human lenses, while found to be completely oxidized in grade II to V cataractous human lenses. Similarly, the Cys170 in lengsin was detected in grade III and above cataractous human lenses (Table 1). The Cys326 in vimentin was 26 times oxidized in LEGSKO vs. age‐matched WT mice lenses (Table 2).

In addition, we also found microfilaments and microtubule proteins that were involved in disulfide cross‐linking, such as actins and tubulins. These cytoskeleton proteins are important components in lens cell structure and organization. It has been shown that disrupting actin assembly by UV light or cytochalasin D promotes cataracts in rat lens (Mousa *et al*., 1979), and interferes with tubulin disulfide bond formation by selenium. This was proposed as a mechanism in cataract formation in the selenium cataractogenesis animal model (Leynadier *et al*., 1991). As illustrated in Fig. 3D, Cys285 motif in gamma‐ and beta‐actin (ACTG1, ACTB, and ACTBL2) was not detected in aged normal and grade II cataractous human lenses, while found significantly oxidized in grade III to V cataractous human lenses. The Cys217 in gamma‐ and beta‐actin (ACTG1 and ACTB) was mildly oxidized in aged normal human lenses, but significantly oxidized in cataractous lenses and associated with severity of cataractous grade. Similar results were seen in LEGSKO lens vs. age‐matched WT lenses and *in vitro* modeling of H_2_O_2_ oxidized lens protein extract vs. nonoxidized controls (Fig. 3E).

We found twofold elevation of the Cys347 in tubulin in LEGSKO lenses compared to age‐matched WT lenses, but no tubulin proteins were detected in human lens samples (Tables 1 and 2). The lens capsule component proteins, such as various type IV collagen chains, were mostly detected in mouse lenses and *in vitro* oxidation. This might be due to the fact that the entire mouse lens was used in our study, while only the lens nucleus was used in the human studies. Strikingly, several cysteine residues in type IV collagen were dramatically oxidized in the LEGSKO compared to WT lenses (Fig. 3F), indicating that oxidation affects not only the lens body but also the lens capsule during aging.

### Disulfide cross‐linking affects a large number of enzymes

**Figure 4:**
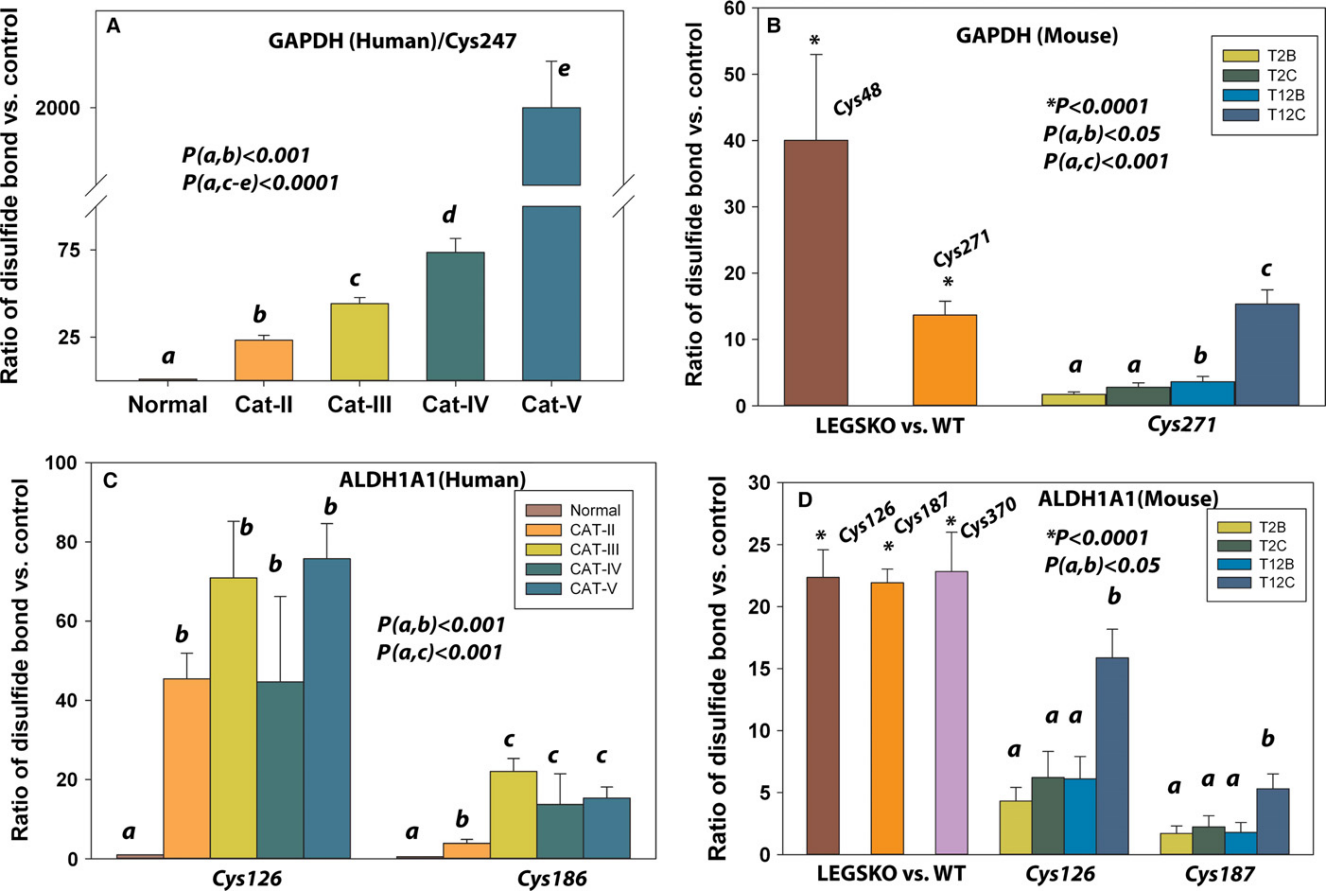
Representative enzymes whose cysteine residues are involved in disulfide cross‐linking. (A) The disulfide bond ratio of the Cys247 in GAPDH in human lens. (B) The disulfide bond ratio of the Cys271 in GAPDH in both LEGSKO mouse and *in vitro* oxidation by H_2_O_2_ compared to their control. In addition, Cys48 in GAPDH was also found close to 40 times oxidized in LEGSKO vs. age‐matched WT mice lenses. (C) The disulfide bond ratio of the Cys126 and Cys186 in ALDH1A1 in human lens. (D) The Cys126 and Cys187 (same motif as human Cys186) were found significantly oxidized in LEGSKO vs. WT and also positively associated with *in vitro* oxidation conditions. In addition, Cys370 oxidation was also detected in LEGSKO mice lenses. See Fig. 3 legend for labels. All ICAT data are expressed as the ratio vs. young lenses pool, LEGSKO vs. age‐matched WT, or oxidized vs. nonoxidized lens protein extract. Standard errors were calculated from three biological replicates. One‐way ANOVA was used to compare the significance between groups, and *P* < 0.05 was considered significant. The significance level was marked by either “*, **“ or lower case letters and the significance value was shown inside the figures.

As we have stated above, a large number of enzymes were identified, including hydrolase, deaminase, small GTPase, nucleotide phosphatase, isomerase, peptidase, ligase, oxygenase, dehydrogenase, cysteine protease, kinase, and reductase. Glyceraldehyde‐3‐phosphate dehydrogenase (GAPDH) was well detected in all three sets of sample pools. The Cys247 located in the catalytic domain of GAPDH in human lens and its similar motif Cys271 in mouse lens were found involving in disulfide bond formation. The oxidation of Cys247 in human lenses was not detectable in aged normal lens. However, significant oxidation was observed in cataractous lenses, and Cys247 was highly oxidized (ratio >1000) in grade V cataractous lenses (Fig. 4A). The homologous motif with Cys271 was over 13‐fold oxidized compared to age‐matched WT lenses and positively associated with oxidation conditions by H_2_O_2_ in *in vitro* modeling (Fig. 4B). In addition, the Cys48 located at NAD binding domain of GAPDH was also identified in disulfide bond formation in LEGSKO mouse lens (Fig. 4B). GAPDH is a highly conserved enzyme that is responsible for an energy‐yielding step in carbohydrate metabolisms, that is, glycolysis. Over the years, more functions have been awarded to this unique protein, such as oxidative stress response, apoptosis, DNA integrity maintenance, intracellular membrane trafficking, autophagy, and cell signaling. There are three cysteine residues present in GAPDH, Cys152, Cys167, and Cys247. It has been indicated that oxidation can inactivate GAPDH activity and subsequently form aggregation, which is most likely due to disulfide cross‐linking (Cumming & Schubert, 2005). This has also proved to be the case in cataract lens (Yan *et al*., 2006).

Another well‐known enzyme in the lens that has been identified from this study is aldehyde dehydrogenase 1, family A1 (ALDH1A1), a family member of NAD(P)+‐dependent enzyme that catalyzes the oxidation of aldehyde to carboxylic acids. We found that Cys126 and Cys186 in human lenses, Cys126, Cys370, and Cys187 (homologous motif with human Cys186) in LEGSKO mouse lenses, and Cys126 and Cys187 in mouse lens protein *in vitro* oxidation were involved in disulfide bond formation. Neither Cys126 nor Cys186 oxidation and disulfide bond were detected in aged normal human lenses, while significant oxidation was found in aged cataractous human lenses in the range of 3‐ to 75‐fold as indicated in Fig. 4C‐D. Over 20‐fold oxidation in these cysteine residues was found in LEGSKO compared to WT mouse lenses. A positive association with *in vitro* oxidation conditions, that is, H_2_O_2_ concentration and incubation time, was also found in the oxidation of these cysteine residues (Fig. 4D).

Several other interesting enzymes were also identified, including aldose reductase (Cys200 in AKR1B1) and sorbitol dehydrogenase (Cys179, Cys140, Cys168 in SORD) in human lenses (see Table 1). In particular, several SORD cysteine residues were completely oxidized in higher‐grade cataractous lenses (Table 1). Similar results were found in phosphoglycerate kinase 1 (PGK1) at its 108th cysteine residue (Table 1). Several enzymes oxidized in identical motifs were detected in all three sets of sample pools, including ubiquitin C‐terminal hydrolase (UCHL1, Cys220) and pyruvate kinase (PKM, Cys123 and Cys400 in human, and homologous motif of Cys49 and Cys326 in mouse). The transketolase (TKT, Cys386) and triosephosphate isomerase 1 (TPI1, Cys218 in human, same motif of Cys268 in mouse) were involved in disulfide bond formation in both human and LEGSKO mouse lenses. Interestingly, Maithal and Shahul *et al*. (Maithal *et al*., 2002) reported that the Cys218 oxidation could abolish TPI activity.

### Disulfide cross‐linking affects antioxidative defense proteins

**Figure 5:**
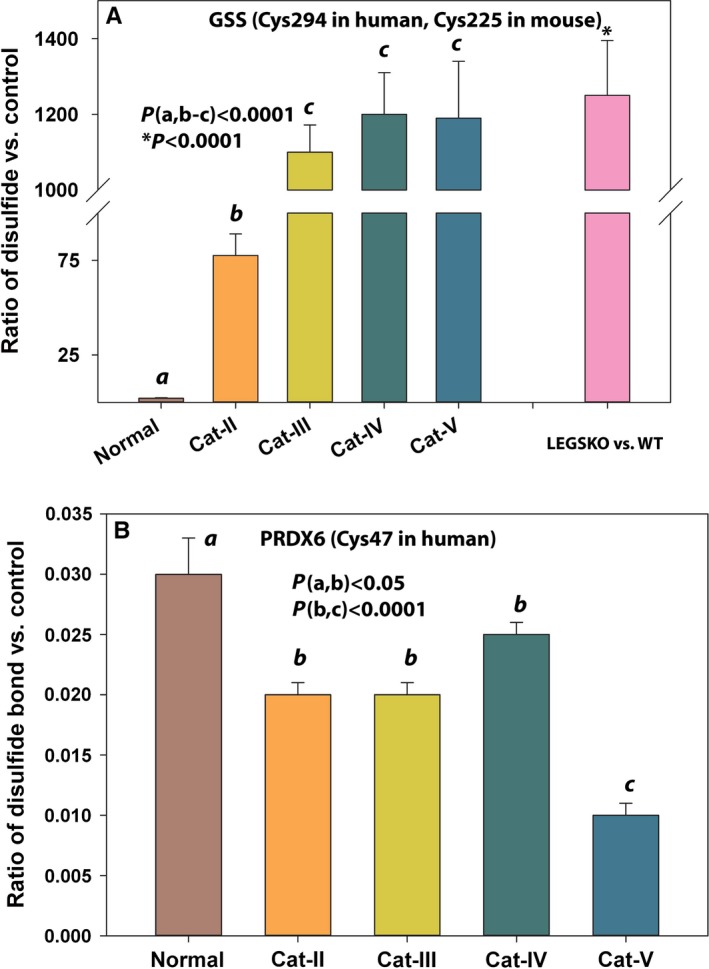
Disulfide cross‐linking affects lens antioxidative proteins. (A) The disulfide bond ratio in of the Cys294 in glutathione synthase (GSS) in human lens. (B). The disulfide bond ratio of the Cys47 in PRDX6 in human lens. See Fig. 3 legend for labels. All ICAT data are expressed as the ratio vs. young lenses pool. Standard errors were calculated from three biological replicates. One‐way ANOVA was used to compare the significance between groups, and *P* < 0.05 was considered significant.

**Figure 6:**
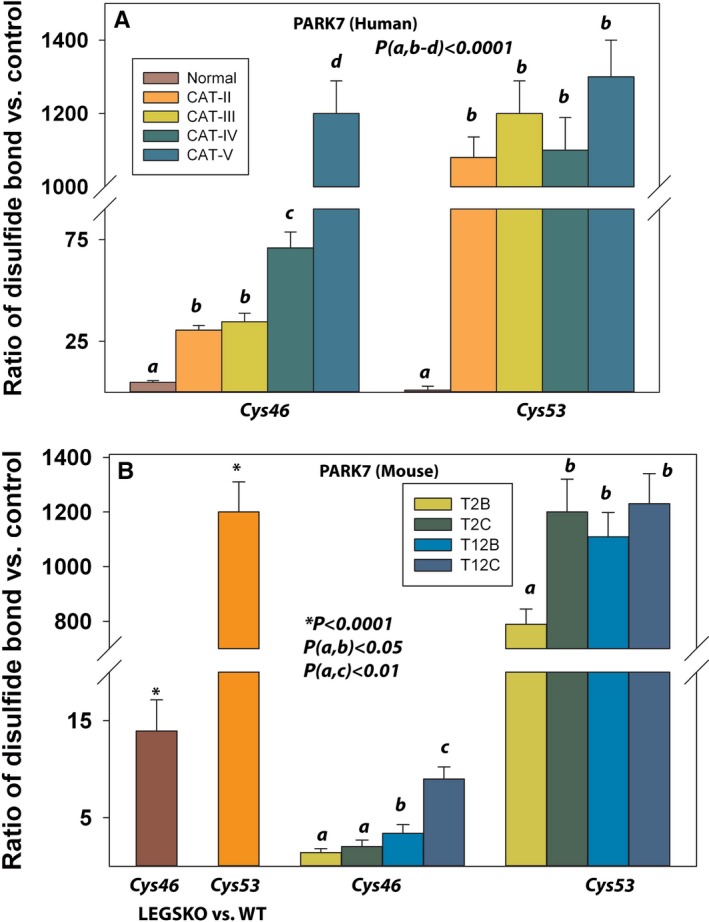
PARK7 (DJ‐1) protein was actively involved in disulfide cross‐linking. (A) The disulfide ratio of the Cys46 and Cys53 in human lens. (B) Similar results were also found in Cys46 and Cys53 oxidation in LEGSKO vs. WT and *in vitro* modeling samples. See Fig. 3 legend for labels. All ICAT data are expressed as the ratio vs. young lenses pool, LEGSKO vs. age‐matched WT, or oxidized vs. nonoxidized lens protein extract. Standard errors were calculated from three biological replicates. One‐way ANOVA was used to compare the significance between groups, and *P* < 0.05 was considered significant.

Aldehyde dehydrogenase is considered as a lens antioxidative protein as described above. We have also found that other proteins involved in disulfide bond formation may have a significant impact on lens antioxidative status, such as glutathione synthase (GSS) and peroxiredoxin (PRDX6). GSS is one of the glutathione *de novo* synthesis enzymes. We found that its 294th cysteine residue (Cys225 in mouse) was moderately oxidized in aged normal human lenses and significantly oxidized in grade II cataractous human lenses, while drastically (ratio>1000) oxidized in grade III and above cataractous human lenses as illustrated in Fig. 5A. Strikingly, this cysteine residue (Cys225) was also strongly oxidized in LEGSKO vs. WT mice lenses (Fig. 5A). However, we did not find modifications of GSS during *in vitro* oxidation. As for PRDX6, we surprisingly found much higher (30‐ to 100‐fold) oxidation/disulfide bond formation at Cys47 in young human lenses compared to aged normal and cataractous human lenses as indicated in Fig. 5B. However, no oxidation was detected in the LEGSKO/WT mouse lens or in the *in vitro*‐oxidized samples. The oxidative stress sensor, Parkinsonism‐associated deglycase (DJ‐1/PARK7), was also detected in all our samples. As illustrated in Fig. 6A,B, Cys46 and Cys53 were oxidized in aged normal and aged cataractous human lenses. Both cysteine residues were also found significantly oxidized in LEGSKO lenses and *in vitro* oxidation by H_2_O_2_ when compared to their controls.

## Discussion

The above data represent to our knowledge the first comprehensive and comparative survey of noncrystallin disulfide bonds forming in age‐related human cataract and a mouse model of low antioxidant defense (the LEGSKO mouse) similar to that found in the nuclear region of the human lens. A major strength of this study is that many of the oxidation sites could be modeled *in vitro* by oxidation with H_2_O_2_ and can be related to the lens crystallin oxidation sites recently published by us (Fan *et al*., 2015). The pending question however is: What is the meaning of these findings and how do the detected oxidation sites relate to the work by others? In particular, the ICAT method used in this work does not allow us to attribute the observed disulfides to protein–protein or mixed disulfides (PSSG and PSSC), with, for example, glutathione and cysteine, which have been well documented to increase in aged human lenses (Lou, 2003). In addition, while the method is powerful for comparison of relative changes in oxidized proteins, it is limited in that it does not provide information on the percentage of oxidation at a particular site. Thus, additional studies for each of the main targets identified will be needed to understand the physiological meaning of the findings.

Of importance, however, is that the majority of proteins involved in disulfide cross‐linking are those with enzymatic functions, which constitute at least one‐third of the proteins identified in this study. The key question is whether the cysteine residue(s) oxidation affects the enzymatic activity of the protein. Indeed, the aging lens has been associated with a declining activity in several enzymes, such as lactate dehydrogenase (LDH) (Zhu *et al*., 2010), ubiquitin conjugation enzymes (Shang *et al*., 1997), and thiol repairing enzymes (Wei *et al*., 2015), such as glutathione reductase (GR), thioredoxin reductase (TR), and glyceraldehyde 3‐phosphate dehydrogenase (GAPDH).

### GAPDH oxidation

GAPDH is known to be prone to inactivation by oxidant stress. It has been extensively studied in the lens and found to be inactivated in aging and cataractous lens, and also by hyperbaric oxygen (HBO) and photooxidation (Padgaonkar *et al*., 1989; Xing & Lou, 2002). It plays an enormous role in controlling glycolysis rate and thus cellular homeostasis as various lens ATP‐dependent biological functions are relying on ATP production by GAPDH. Yan *et al*. (2006) found thioredoxin (Trx) and thioredoxin reductase (TrxR) were able to revive inactivated GAPDH in aged normal and cataractous human lenses. These results clearly indicate that disulfide formation must be in part the trigger causing protein conformational change or blocking the cysteine site in or close to the active site. The impact of weakened GAPDH activity might also be responsible for the accumulation of methylglyoxal‐derived advanced glycation end products, as proposed by Brownlee (Brownlee, 2001). This may result in a paradoxical improvement of chaperone activity of αA‐crystallin (Kanade *et al*., 2009), while methylglyoxal itself can cause mitochondrial dysfunction, ER activation, and lens epithelial cell apoptosis (Palsamy *et al*., 2014).

### Aldehyde dehydrogenase oxidation

Another metabolic enzyme hit by oxidation was aldehyde dehydrogenase. In the eye, this enzyme is described as both ‘corneal and lens crystallin’ (Marchitti *et al*., 2011). In addition, Lassen *et al*. (2007) have reported that ALDH1A1 may also have an antioxidative function, that is, prevent UV‐induced damage. In the same study, ALDH1A1 knockout mice were found developing cataracts at 6–9 months of age especially sensitive to UV light. Interestingly, the mechanism of inhibition of aldehyde dehydrogenase by disulfiram, a drug used to treat chronic alcoholism, was found actually to involve the disulfide formation (Vallari & Pietruszko, 1982).

### Oxidation of redox enzymes

Equally important are those enzymes involved in controlling the redox state of the lens such as PRDX6 and GSS. The Cys294 was highly oxidized in cataractous human lenses, and a similar result was also found in LEGSKO mice lenses (Cys225), although the GSS peptides found from *in vitro* modeling did not make the Mascot score threshold possibly due to loss in the insoluble fraction. In addition, due to either low abundance or mass spectrometry sensitivity, a large number of proteins that were identified by ICAT could not be quantitatively determined due to lack of dimethyl‐labeled peptides. This may also explain why we did not report some of mitochondrial electron transporter chain‐related enzymes, which have been reported prone to oxidation (Musatov & Robinson, 2012; Wu *et al*., 2014). On the other hand, it has also been reported (Xing & Lou, 2010) that some of the lenticular enzymes are more resistant to oxidative stress, such as thioredoxin reductase and glutathione reductase. This might also explain why some key redox enzymes were missing from this study. Future work is needed to verify whether disulfide bond formation by the cysteine residue may affect GSH *de novo* synthesis.

Quite in contrast to GSS, we found 30 to 100 times less disulfide bond formation at Cys47 in PRDX6 in aged normal and aged cataractous human lenses compared to the young lens pool. The peroxidase active site of PRDX6 motif is PVC47TT. The Cys47 is actively engaged in oxidation, disulfide formation, and regeneration by GSH to detoxify its substrates, that is, H_2_O_2_, short‐chain organic, fatty acid, and phospholipid hydroperoxides. So, it is likely that lower disulfide bond formation is linked to impaired PRDX6 redox activity due to Cys47 oxidation. As a consequence, the inability to engage with its substrate may result in loss of its detoxification function in aged and cataractous lens. Taken together, these results suggest that the aging lens is chronically moving into a vicious cycle whereby lens protein damage by oxidation further enhances imbalance of the redox state.

### Oxidation of cytoskeleton proteins

Major oxidative changes were also observed in cytoskeleton proteins. It is well documented that these proteins play a critical role in lens transparency (Quinlan *et al*., 1999). In our study, all three major cytoskeleton proteins, microfilaments, microtubules, and intermediate filaments were found heavily involved in disulfide bond formation in aged, GSH‐deficient, and cataractous lenses. IFs are particularly important for lens structure due to the lens’ unique high order of cell–cell organization, unusually high protein content (reach to 400 mg mL^−1^ in the lens nucleus region). Lens‐specific IF proteins, beaded filament structural proteins 1 and 2 (BFSP1 BFSP2), also known as filensin and phakinin, through their interactions with chaperones, guide the lens cellular organization and alignment to achieve its high refractive index. The importance of BFSP1 and BFSP2 was further evidenced by their mutation‐associated cataract phenotype (Conley *et al*., 2000). Besides these, other lens structure component proteins were also oxidized, including fibrillin‐1 (FBN1), plectin (PLEC), spectrin alpha (SPTAN1), and lens intrinsic membrane protein 2 (LIM2). FBN1 is an important component of lens zonules, and mutation of FBN1 has been found to be associated with ectopia lentis (Khan *et al*., 2014). Plectin has been found associated with intermediate filament proteins (Wiche & Winter, 2011), and LIM2 is an eye‐specific protein found at the junctions of lens fiber cells. However, whether the oxidation and disulfide bond formation in these cysteine residues will cause protein conformational change and ultimately alter lens cytoskeleton organization and aggregate formation remains to be clarified.

### Oxidation of other vital proteins

Finally, one interesting question is the extent to which the protein cysteine oxidation data obtained in lens are applicable to other cellular systems. Clearly, lens proteins are long‐lived proteins and therefore prone to accumulation of damage. Neurons, on the other hand, are postmitotic cells prone to neurodegeneration and similar accumulation of protein damage. In that regard, Parkinsonism‐associated deglycase (DJ‐1/PARK7) that was detected in all our samples is not only an oxidative stress sensor (van der Merwe *et al*., 2015), but also a chaperone that inhibits protein aggregation, such as α‐synuclein (Shendelman *et al*., 2004). PARK7 has also been reported highly expressed in lens fibers (Sun *et al*., 2015). There are three cysteine residues in PARK7, Cys46, Cys53, and Cys106. The Cys106 is a critical PARK7 antioxidant mediator. Under oxidative stress, Cys106 will be sulfinated or sulfonated to shift to a more acidic isoform triggering an oxidation elimination response. The Cys46 and Cys53 have much less regulatory activity than Cys106 (Meulener *et al*., 2005), but Cys53 was found to be playing an important role in the stabilization of PARK7, so the Cys106 can form heterodisulfide protein complexes (Fernandez‐Caggiano *et al*., 2016). We did not detect Cys106 disulfide in PARK7, but dramatic oxidation was found in Cys46 and Cys53 implying that Cys106 may have been hyperoxidized due to PARK7 protein conformational change via extensive oxidation of Cys46 and Cys53 residues.

In summary, in agreement with the outcome of the crystallin protein cysteine residue oxidation studies in the same lens extracts (Fan *et al*., 2015), the oxidation pattern of cysteine residues in other lens proteins was reproducible and found to share very similar pattern from human to mouse and between *in vitro* and *in vivo* conditions. These results further support the important role of oxidant stress in age‐related cataract formation, and in particular the importance of GSH homeostasis as all three systems under investigation were characterized by low (in the lens nucleus) to absent GSH. From a therapeutic perspective, the recapitulation of the protein chemistry changes via *in vitro* modeling indicates that pharmacological GSH mimetics are needed to protect the aging lens from oxidation. In that regard, the above work represents, to our knowledge, the first comprehensive database on protein disulfides of noncrystallin proteins in aged and cataractous lenses.

## Materials and methods

### Animals

All animal experiments were conducted in accordance with procedures approved by the Case Western Reserve University Animal Care Committee and conformed to the ARVO Statement for Use of Animals in Ophthalmic and Vision Research. Animals were housed under diurnal lighting condition and allowed free access to food and water. Lens conditional γ‐glutamyl cysteine ligase catalytic subunit (Gclc) knockout mice were created as previously described by us and named as LEGSKO mouse (20).

### Human and mouse lens samples

All human tissues used for this study were approved by Case Western Reserve University and Sun Yat‐sen University Institutional Review Board (IRB). Three types of human lens nuclei were used in this study after careful removal of ~1mm cortical layer. Three young normal lens nuclei were pooled to serve as control for entire human lens study. The young and aged normal lenses at age 3, 7, 15, 68, 72, and 74 years were collected from the Cleveland Eye Bank/Midwest Eye Bank within postmortem interval of 2–8 h (average, 4.1 h). Human cataract lenses from patients undergoing extracapsular cataract extraction (ECCE) surgery were collected from Huichang County People's and Ganzhou City People's Hospital in Jiangxi, China, after obtaining informed consent from the patients. The cataract severity was graded based on Lens Opacities Classification System (LOCS) III. Five grades were given (from I to V) to categorize the severity of cataract with higher grade corresponding to higher severity. Only grade II to V lenses were collected in this study. The ages of the grade II group were 64, 67, and 60 years; the grade III were 60, 59, and 69 years; the grade IV were 56, 63, and 77 years; and the grade V were 75, 80, and 78 years. All cataract lenses were processed within 30 min after surgery to prevent artefactual oxidation by homogenization in 10% trichloroacetic acid (TCA) and 100 mm iodoacetamide (IAM) immediately after receipt. The lens precipitate was then resuspended in freshly prepared ICAT buffer (200 mm Tris–HCl, pH 8.5, 6 m urea, 5 mm EDTA, 0.05% SDS, and 100 mm IAM) to further block free sulfhydryl group. All processed human lens samples collected in China were shipped to Cleveland for ICAT labeling and proteomics study after initial blocking of the free sulfhydryl groups. The details on lens nucleus isolation and pre‐ICAT preparations are described in our previous report (Fan *et al*., 2015). For the mouse lens study, 9‐month‐old LEGSKO whole lenses with nuclear cataract and age‐matched WT whole lenses were used. Three age‐matched wild‐type whole lenses were pooled to serve as control for the LEGSKO cataract lenses.

### *In vitro* modeling of lens protein disulfide cross‐linking

The mouse lens protein extract from 3‐month‐old lenses was incubated with or without 1 or 5 mm hydrogen peroxide (H_2_O_2_) in Chelex‐treated 50 mm potassium phosphate buffer (pH 7.4) containing 1 mm DTPA at 37 °C for up to 12 h. The samples at 2 and 12 h were taken and processed the same way as human lenses as described above.

### Quantitative Isotope‐coded Affinity Tag labeling Experiment (ICAT)

For ICAT‐based quantification by mass spectrometry, 100 μg of protein aliquot of lens sample was reduced by 10 mm *tris*‐(2‐carboxyethyl)phosphine (TCEP) before labeling by cleavable ICAT reagent (AB Sciex, Foster City, CA, USA) as described in the instruction manual. All human aged normal and aged cataract and LEGSKO mouse cataract lens samples were labeled with the cleavable heavy ICAT reagent (^13^C‐ICAT), whereas the young human and age‐matched wild‐type mouse lens control samples were labeled with the cleavable light ICAT reagent (^12^C‐ICAT). After labeling, a 1:1 mixture of each aged or cataract lens sample with control sample was prepared. The proteins were digested sequentially by lysyl endopeptidase (Wako, Richmond, VA, USA) and trypsin (Promega, Madison, WI, USA), and the peptides were purified sequentially through Oasis HLB Plus column (Waters, Milford, MA, USA) and POROS 50HS column (Thermo Fisher, Grand Island, NY, USA). The disulfide bonding peptides were enriched and further purified by Avidin affinity column (Thermo Fisher). The biotin group in dried sample was removed by trifluoric acid (TFA) before LC‐MS analysis. A more detailed description of ICAT labeling and purification procedures can be found in our previous report (Fan *et al*., 2015).

### Quantification of proteins by dimethyl labeling

The purpose of the dimethyl labeling was to normalize the ICAT ratios to adjust for differences in the abundance of the cysteine containing proteins between samples. Aliquots (100 μg) from same original pool of samples that were subjected to the ICAT labeling were digested, and the peptides were purified as described in our previous report (Fan *et al*., 2015). The tryptic peptides from control sample were labeled with normal formaldehyde and tryptic peptides from aged and cataract samples were labeled with deuterium‐labeled formaldehyde (Sigma‐Aldrich, St. Louis, MO, USA), respectively, according to previous report (Garcia‐Santamarina *et al*., 2011). After labeling, a 1:1 mixture of each aged or cataract lens sample with control sample was prepared and purified for LC‐MS/MS study.

### Mass spectrometry (MS) analysis of ICAT‐ and dimethyl‐labeled samples

Peptides were analyzed by liquid chromatography–tandem mass spectrometry (LC‐MS/MS) using Orbitrap Elite Hybrid Mass Spectrometer (Thermo Electron, San Jose, CA, USA) coupled with a Waters nanoAcquity UPLC system (Waters, Taunton, MA, USA). The peptides were separated using a linear gradient of acetonitrile in 0.1% formic acid from 2% to 35% for 60 min and then up to 90% for 15 min prior to column equilibration with water in 0.1% formic acid for 15 min, making a total run‐time of 90 min. The spectra were acquired in the positive ionization mode by data‐dependent methods consisting of a full MS scan in high‐mass accuracy FT‐MS mode at 120 000 resolution, and MS/MS on the twenty most abundant precursor ions in CID mode with the normalized collision energy of 35%. Mascot Daemon (version 2.4.0; Matrix Science) was used to identify the peptides, and the data were searched against SwissProt human or mouse database. The mass tolerance was set at 10 ppm for precursor ions and 0.8 Da for product ions. For dimethyl labeling, the light (with regular formaldehyde)‐ and intermediate (with deuterated formaldehyde)‐labeled peptides at their N‐terminal and Lys residues (with the mass shift of 28.0313 and 32.0475 Da, respectively) in addition to carbamidomethylation of Cys and oxidation of Met residues were set at as variable modifications for Mascot Demon. Excluding crystallin peptides, peptides with light labeling and intermediate or heavy labeling in each protein were analyzed and the ratio of light to heavy dimethylation modification was calculated by manual extraction of each peptide on the basis of peptide peak heights. For ICAT labeling, the light and heavy ICAT‐labeled peptides with mass shift of 227.1270 and 236.1571 Da, respectively, were first identified through Mascot Demon and then confirmed via manual examinations. The average ratio from multiple spectra covering the same sites of particular peptide was calculated in each sample, and the average ratio from three samples after adjustment from dimethyl labeling results was reported as final ICAT quantification results. For both ICAT‐ and dimethyl‐labeled samples, Mascot ions score threshold is set at 20. The false discovery rate of peptide matching is identified within 3%. The detailed procedures of ICAT‐labeled peptide identification and quantification have been reported in our previous study (Fan *et al*., 2015). All reported ICAT ratios were identified with the standard error < 0.1 and correlation coefficiency > 0.99 as described in previous report (Leung *et al*., 2005). The mass spectrometry proteomics data have been deposited to the ProteomeXchange Consortium via the PRIDE partner repository with the dataset identifier PXD002658 and 10.6019/PXD002658.

### Statistical methods

All values were expressed as means ± SE. Statistical analysis was performed according to the methods previously described in detail in Sell *et al*. (2000). In brief, statistical significance of differences in mean values was assessed by repeated‐measures ANOVA or Student's *t*‐test. *P*‐values of < 0.05 were considered statistically significant.

## Funding

This research was supported by grants EY07099 (VMM) and EY024553 (XF) and Case Western Reserve University Visual Science Research Center (NEI P30EY‐11373).

## Author contributions

XF and VMM conceived the project. XF, BW, GH, and SZ conducted the experiments, and MG, BL, and JY supplied the essential materials. XF, BW, and VMM interpreted the data. XF and VMM wrote the manuscript.

## Conflict of interest

None declared.

## Supporting information


**Fig. S1.** To quantitatively determine cysteine disulfide bond formation by isotope‐coded affinity tag (ICAT) and dimethyl labeling proteomics approach.
**Fig. S2.** Representative mass spectrum of ICAT labeling.
**Fig. S3.** Representative mass spectrum of dimethyl labeling.
**Table S1.** Amount of disulfide bonding in peptides from proteins of mouse lens protein extract oxidized by hydrogen peroxide.
**Table S2.** Dimethyl (regular formaldehyde) and intermediate (deuterated formaldehyde) labeled peptides identified from human lens ICAT samples by mass sectrometry (MS).
**Table S3.** Dimethyl (regular formaldehyde) and intermediate (deuterated formaldehyde) labeled peptides identified from mouse lens ICAT samples by mass sectrometry (MS).
