# Supplementary material for: The oxidized thiol proteome in aging and cataractous mouse and human lens revealed by ICAT labeling
Source: Aging Cell. 2016 Nov 13;16(2):244–61. doi: 10.1111/acel.12548 (PMC5334568; doi:10.1111/acel.12548)
Supplement: Supplementary file 1 — Fig. S1. To quantitatively determine cysteine disulfide bond formation by isotope‐coded affinity tag (ICAT) and dimethyl labeling proteomics approach. Fig. S2. Representative mass spectrum of ICAT labeling. Fig. S3. Representative mass spectrum of dimethyl labeling. Table S1. Amount of disulfide bonding in peptides from proteins of mouse lens protein extract oxidized by hydrogen peroxide. Table S2. Dimethyl (regular formaldehyde) and intermediate (deuterated formaldehyde) labeled peptides identified from human lens ICAT samples by mass sectrometry (MS). Table S3. Dimethyl (regular formaldehyde) and intermediate (deuterated formaldehyde) labeled peptides identified from mouse lens ICAT samples by mass sectrometry (MS). [file ACEL-16-244-s001.pdf]

Table S1. Amount of disulfide bonding in peptides from proteins of mouse lens protein extract oxidized by hydrogen peroxide.

Protein Name	Description	Peptide Sequence[a]	$ICAT_{OX}/ICAT_{non-ox}$[b,c,d,e]			
			T2B	T2C	T12B	T12C
**Catalytic Activity**						
TPI1	triosephosphate isomerase 1	IAVAAQNC117YK	1.88(±0.23)	2.13(±0.11)	2.37(±0.32)	8.46(±0.48)
MTAP	methylthioadenosine phosphorylase	TSLRPQTFYDGSHC130SAR	5.35(±0.61)	5.47(±0.45)	12.31(±1.32)	17.69(±3.48)
GCLC	glutamate-cysteine ligase, catalytic subunit	GGNAVVDGC501SK	2.75(±0.98)	1.66(±0.37)	10.72(±2.78)	54.12(±10.27)
PARK7	Parkinsonism associated deglycase	DPVQC46SR	1.41(±0.11)	2.05(±0.32)	3.77(±0.33)	8.91(±0.72)
		DVVIC53PDASLEDAKK	800.23(±63.19)	>1000	>1000	>1000
EEF1A1	eukaryotic translation elongation factor 1 alpha 1	SGDAAIVDMVPGKPMC411VESFSDYPPLG R	1.90(±0.22)	2.45(±0.25)	3.44(±0.51)	5.17(±0.71)
		DGSASGTTLLEALDC234ILPPTRPTDKPLR	ND	ND	3.72(±0.44)	8.26(±2.17)
EIF4A1	eukaryotic translation initiation factor 4A1	AILPC66IK	ND	ND	2.57(±0.61)	7.22(±2.08)
AKR1B1	aldo-keto reductase family 1, member B1	HIDC45AQVYQNEK	2.46(±0.23)	3.17(±0.77)	6.12(±1.79)	10.60(±2.42)
GAPDH	glyceraldehyde-3-phosphate dehydrogenase	VPTPNVSVVDLTC271R	1.91(±0.24)	2.7(±0.17)	3.62(±0.37)	14.98(±2.04)
ENO1	enolase 1	VNQIGSVTESLQAC357K	1.17(±0.31)	2.06(±0.57)	2.77(±1.02)	17.30(±2.47)
EEF2	eukaryotic translation elongation factor 2	YVEPIEDVPC466GNIVGLVGVDQFLVK	ND	ND	2.44(±0.31)	4.89(±0.42)
PRDX1	peroxiredoxin 1	HGEVC173PAGWKPGSDTIKPDVNK	2.48(±0.77)	1.37(±91)	2.42(±0.37)	5.39(±0.95)
PHGDH	3-phosphoglycerate dehydrogenase	NAGTC369LSPAVIVGLLR	ND	ND	3.01(±0.81)	6.25(±0.34)
		ALVDHENVISC281PHLGASTK	ND	ND	ND	3.93(±0.69)
UBE2O	ubiquitin-conjugating enzyme E2O	VQSC568PDPAVYGVVQSGDHVGR	1.12(±0.34)	1.91(±0.77)	3.02(±0.51)	4.27(±1.02)
		LYDVC239PHVSDSGLFFDDSYGFYPGQVLI GPAK	ND	ND	3.05(±0.70)	9.05(±1.84)
ASS1	argininosuccinate synthase 1	FELTC132YSLAPQIK	3.11(±0.24)	1.91(±039)	2.41(±0.98)	4.83(±1.22)
PEPD	peptidase D	GVNTDSGSVC158R	2.21(±0.35)	4.64(±0.32)	8.94(±1.10)	25.06(±2.04)
LGSN	lengsin	TNMFC383SGSGVER	8.79(±2.47)	17.09(±5.62)	10.13(±1.73)	12.75(±3.58)
		ATC196FNSDIVLMPELSTFR	ND	ND	6.11(±2.33)	67.11(±7.73)
		DLKDSVPTTWGYNDNSC445ALNIK	ND	ND	3.88(±0.72)	12.59(±2.14)
ADH5	alcohol dehydrogenase 5	AKEFGASEC240ISPQDFSK	23.71(±4.49)	>1000	>1000	>1000
PAICS	phosphoribosylaminoimidazole carboxylase	ITSC63IFQLLQEAGIK	1.77(±0.44)	3.64(±0.52)	3.57(±0.81)	5.28(±0.77)
ALDH1A1	aldehyde dehydrogenase family 1, subfamily A1	IGPALSC187GNTVVVKPAEQTPLTALHLAS LIK	2.24(±0.63)	1.71(±0.47)	2.53(±0.62)	5.27(±0.57)
		VFANAYLSDLGGC126IK	4.32(±0.47)	6.03(±0.35)	5.97(±0.49)	17.45(±1.33)
DPYSL2	dihydropyrimidinase-like 2	SITIANQTNC248PLYVTK	1.72(±0.20)	3.21(±0.44)	8.99(±1.19)	35.84(±2.77)
UCHL1	ubiquitin C-terminal hydrolase L1	FSAVALC220K	3.75(±0.11)	7.27(±0.44)	12.13(±0.89)	74.07(±10.41)

		NEAIQAAHDSVAQEGQC152R	7.27(±0.60)	14.43(±0.79)	6.74(±3.23)	105.26(±17.4)
PPIA	peptidylprolyl isomerase A	IIPGFMC62QGGDFTR	1.11(±0.17)	1.19(±0.09)	2.25(±0.33)	9.05(±1.17)
FN3KRP	fructosamine 3 kinase related protein	ATGHSGGGC24ISQGQSYDTDK	2.11(±0.47)	2.31(±0.39)	2.77(±0.61)	5.06(±0.72)
PKM	pyruvate kinase, muscle	GIFPVLC474K	1.44(±0.17)	1.83(±0.33)	1.85(±0.50)	4.90(±0.79)
		NTGIIC49TIGPASR	1.82(±0.46)	1.97(±0.30)	4.07(±0.62)	4.07(±0.77)
		AGKPVIC326ATQMLESMIK	1.55(±0.31)	2.49(±0.44)	4.21(±0.74)	7.21(±1.17)
		AEGSDVANAVLDGADC358IMLSGETAKG DYPLEAVR	ND	ND	5.05(±1.72)	15.33(±2.94)
Strucutre molecule activity						
RPS28	ribosomal protein S28	TGSQGQC28TQVR	2.17(±0.18)	2.97(±0.44)	2.83(±0.31)	6.54(±2.37)
SPTAN1	spectrin alpha, non-erythrocytic 1	GAC1627AGSEDAVK	1.22(±0.41)	1.76(±0.79)	1.42(±0.63)	2.74(±0.91)
PDLIM1	PDZ and LIM domain 1	LPIC258DK	2.78(±0.63)	1.45(±0.92)	2.09(±0.86)	4.21(±0.58)
VIM	vimentin	QVQSLTC328EVDALK	4.81(±0.50)	8.62(±0.39)	8.91(±1.25)	36.10(±4.62)
KRT1	keratin 1	C334EMEQQNQEYK	1.52(±0.71)	2.09(±0.41)	2.52(±0.66)	4.31(±0.56)
KRT6A	keratin 6A	LLEGEEC463R	3.34(±0.23)	3.77(±0.18)	3.56(±0.51)	7.79(±0.40)
ACTG1	actin gamma 1	C163DVDIR	2.58(±0.22)	9.23(±1.75)	11.71(±2.31)	238.12(±47.6)
ACTB	actin, beta	C285DVDIR	1.79(±0.28)	1.70(±0.47)	1.86(±0.91)	3.90(±0.21)
		LC217YVALDFEQEMATAASSSSLEK	1.09(±0.34)	1.15(±0.67)	1.80(±0.31)	8.81(±1.17)
RPSA	ribosomal protein SA	YVDIAIPC163NNK	ND	ND	ND	4.05(±0.61)
		ADHQPLTEASYVNLPTIALC148NTDSPLR	3.23(±0.42)	5.50(±0.77)	4.99(±0.89)	18.91(±4.00)
TUBA1A	tubulin, alpha 1A	TIQFVDWC347PTGFK	1.38(±0.09)	2.22(±0.27)	2.35(±0.66)	4.55(±0.30)
		AVC376MLSNTTAIAEAWAR	1.02(±0.11)	1.09(±0.43)	5.01(±1.01)	19.78(±3.34)
		AYHEQLSVAEITNAC295FEPANQMVK	1.62(±0.67)	1.99(±0.80)	2.07(±0.48)	8.90(±1.27)
LIM2	lens intrinsic membrane protein 2	YC46LGNK	3.69(±0.45)	3.03(±0.55)	3.77(±0.31)	3.39(±0.61)
CAP1	CAP, adenylate cyclase-associated protein 1	LEAVSHTSDMHC29GYGDSPSK	2.77(±0.90)	3.61(±0.73)	3.99(±0.75)	5.34(±0.90)
TUBB2A	tubulin beta 2A class IIa	TAVC354DIPPR	0.08(±0.01)	0.08(±0.01)	0.082(±0.01)	1.0(±0.02)
		NMMAAC303DPR	1.63(±0.11)	1.77(±0.36)	2.04(±0.74)	3.81(±0.32)
		EIVHIQAGQC12GNQIGAK	1.70(±0.20)	1.96(±0.20)	1.78(±0.57)	4.49(±0.22)
		LTTPTYGDLNHLVSATMSGVTTC239LR	3.77(±0.33)	4.51(±0.70)	6.10(±1.04)	23.21(±4.11)
KRT5	keratin 5	LLEGEEC474R	1.58(±0.11)	2.79(±0.09)	7.13(±3.54)	4.88(±1.37)
BFSP1	beaded filament structural protein 1	SSYDC290R	1.50(±0.23)	2.17(±0.31)	3.73(±0.62)	7.24(±0.75)
**Binding (PARK7, EEF1A1, EIF4A1, SPTAN1, PDLIM1, EEF2, UBE2O, PEPD are also belong to this category)**						
EIF5A	eukaryotic translation initiation factor 5A	KYEDIC73PSTHNMDVPNIK	0.74(±0.35)	1.55(±0.11)	1.60(±0.26)	4.49(±0.37)
**Nucleic acid binding transcription factor (PARK7, PDLIM1 and PEPD are in this category)**						
**Antioxidant activity (PRDX1 is also belong to this category)**						
**Translation regulatory (EIF5A, EEF1A1, EIF4A1, EEF2 are also bleong to this category)**						
**Transporter activity (AKR1B1 and LIM2 are also belong to this category)**						

**Other funcations**						
FABP5	fatty acid binding protein 5	TTVFSC67NLGEK	1.00(±0.08)	1.26(±0.21)	1.32(±0.25)	2.63(±0.18)
		TETVC87TFQDGALVQHQQWDGK	1.03(±0.37)	1.03(±0.55)	1.32(±0.14)	2.26(±0.55)
HSPA8	heat shock protein 8	GPAVGIDLGTTYSC17VGVFQHGK	2.81(±0.73)	3.91(±0.30)	2.80(±1.14)	8.19(±2.46)
CLTC	clathrin heavy chain	IHEGC870EEPATHNALAK	4.73(±1.09)	25.25(±4.42)	31.89(±5.21)	62.57(±10.09)
DPYSL2	dihydropyrimidinase like 2	SITIANQTNC248PLYVTK	5.82(±1.48)	11.62(±3.44)	15.01(±5.88)	35.84(±7.14)
HSP90AB1	heat shock protein 90kDa alpha family class B member 1	FENLC564K	3.01(±1.20)	10.04(±3.48)	9.83(±2.72)	44.44(±3.00)
PFN1	profilin 1	C128YEMASHLR	2.22(±0.40)	2.59(±0.39)	2.88(±0.21)	11.40(±3.07)
GLOD4	glyoxalase domain containing 4	AAC45NGPYDGK	2.59(±0.14)	2.02(±0.58)	2.23(±0.70)	2.66(±0.92)
		HEEFEEGC41K	1.17(±0.42)	1.77(±0.39)	1.39(±0.95)	3.94(±0.77)
IDH1	isocitrate dehydrogenase 1	C73ATITPDEK	2.78(±0.62)	3.76(±0.44)	5.19(±0.87)	6.07(±1.04)
ALB	albumin	YIC289ENQDSISSK	1.88(±0.48)	1.98 (±0.52)	1.79(±0.41)	2.32(±0.91)
		RPC511FSALEVDETYVPK	0.96(±0.32)	1.21(±0.46)	0.90(±0.88)	2.36(±0.79)
		LC99AIPNLR	2.23(±0.75)	1.90(±0.75)	2.04(±0.40)	2.13(±0.77)
		TNC416DLYEK	1.03(±0.10)	0.90(±0.34)	1.13(±0.21)	2.35(±0.36)
		DTC591FSTEGPNLVTR	1.00(±0.24)	0.97(±0.50)	1.72(±0.33)	2.44(±0.67)
		VC485LLHEK	1.17(±0.69)	0.94(±0.78)	1.40(±0.30)	2.25(±0.46)
		AETFTFHSDIC535TLPEKEK	1.86(±0.15)	35.71(±4.82)	30.21(±5.29)	84.74(±12.32)
ALDOA	aldolase, fructose-bisphosphate A	ALANSLAC393QGK	5.16(±1.09)	7.15(±2.33)	17.88(±6.20)	66.22(±9.87)
		YASIC232QQNGIVPIVEPEILPDGDHDLKR	1.37(±0.31)	1.96(±0.40)	2.53(±0.26)	5.83(±1.01)

Asterisk denote whether the protein or peptide or both were previously reported.
[a] Amino acid sequence number of the identified peptide in the N to C terminal direction.
[b] This ratio of disulfides in mouse lens protein extract oxidized by H_2O_2 vs. no-oxidized was determined as ICAT-^{13}C/ICAT-^{12}C.
[c] The ICAT ratio has been adjusted based on dimethyl labeling results.
[d] The number in parenthesis is the value of standard error (SE).
[e] ND is no detectable.

Table S2. Dimethyl (regular formaldehyde) and intermediate (deuterated formaldehyde) labeled peptides identified from human lens ICAT samples by mass sectrometry (MS)

GENE I	pep_exp_	pep_calc	pep_d	pep	pep_seq	pep_var_mod
PRDX6	933.4924	933.492	0.0005	25	NFDEILR	Dimethyl (N-term)
	937.5174	937.5081	0.0092	21	NFDEILR	Intermediate Dimethyl (N-term)
	1422.6816	1422.6813	0.0003	45	DFTPVCTTELGR	Carbamidomethyl (C); Dimethyl (N-term)
	1426.706	1426.6975	0.0085	25	DFTPVCTTELGR	Carbamidomethyl (C); Intermediate Dimethyl (N-term)
FAH	1199.6875	1199.6874	0.0001	44	ASSVVVSGTPIR	Dimethyl (N-term)
	1226.7115	1226.7114	0.0001	42	ASSVVVSGTPIR	Intermediate Dimethyl (N-term)
UCHL1	814.4807	814.48	0.0008	26	QIEELK	Dimethyl (K); Dimethyl (N-term)
	822.5308	822.5303	0.0005	36	QIEELK	Intermediate Dimethyl (K); Intermediate Dimethyl (N-term)
	1036.5433	1036.5441	-0.0007	52	QFLSETEK	Dimethyl (K); Dimethyl (N-term)
	1044.5667	1044.5764	-0.0097	22	QFLSETEK	Intermediate Dimethyl (K); Intermediate Dimethyl (N-term)
SERPINB6	978.5526	978.5498	0.0028	50	TGTQYLLR	Dimethyl (N-term)
	982.5751	982.566	0.0091	35	TGTQYLLR	Intermediate Dimethyl (N-term)
	1513.6949	1513.697	-0.0021	35	LEESYDMESVLR	Dimethyl (N-term); Oxidation (M)
	1517.7204	1517.7132	0.0073	24	LEESYDMESVLR	Intermediate Dimethyl (N-term); Oxidation (M)
SERPINB9	1064.544	1064.5437	0.0004	34	ANSILFCGR	Carbamidomethyl (C); Dimethyl (N-term)
	1068.569	1068.5683	0.0006	31	ANSILFCGR	Carbamidomethyl (C); Intermediate Dimethyl (N-term)
PPIA	1625.7695	1625.7694	0.0001	39	IIPGFMCQGGDFTR	Carbamidomethyl (C); Dimethyl (N-term)
	1629.7951	1629.7856	0.0095	35	IIPGFMCQGGDFTR	Carbamidomethyl (C); Intermediate Dimethyl (N-term)
LGSN	858.4526	858.4527	-0.0001	30	FLEYFI	Dimethyl (N-term)
	862.4772	862.4689	0.0083	26	FLEYFI	Intermediate Dimethyl (N-term)
	1258.6312	1258.6306	0.0006	28	FEATDLHGVSR	Dimethyl (N-term)
	1262.6564	1262.6468	0.0096	23	FEATDLHGVSR	Intermediate Dimethyl (N-term)
	2033.0475	2033.0504	-0.0029	79	VICDTFTVTGEPLLTSPR	Carbamidomethyl (C); Dimethyl (N-term)
	2037.0694	2037.0665	0.0029	64	VICDTFTVTGEPLLTSPR	Carbamidomethyl (C); Intermediate Dimethyl (N-term)
GSS	968.6018	968.6018	-0.0001	67	ALAEGVLLR	Dimethyl (N-term)
	972.6276	972.618	0.0096	44	ALAEGVLLR	Intermediate Dimethyl (N-term)
ENO1	959.5175	959.5175	0	23	IEEELGSK	Dimethyl (K); Dimethyl (N-term)

	959.5176	959.5175	0.0001	37	IEEELGSK	Dimethyl (K); Dimethyl (N-term)
	967.5658	967.5657	0.0001	42	IEEELGSK	Intermediate Dimethyl (K); Intermediate Dimethyl (N-term)
SORD	936.5389	936.5392	-0.0004	27	VAIEPGAPR	Dimethyl (N-term)
	940.5644	940.5554	0.009	37	VAIEPGAPR	Intermediate Dimethyl (N-term)
	959.365	959.3655	-0.0005	37	CDPSDQNP	Carbamidomethyl (C); Dimethyl (N-term)
	1977.0566	1977.0571	-0.0005	50	LENYPIPEPGPNEVLLR	Dimethyl (N-term)
	1981.0823	1981.0733	0.009	21	LENYPIPEPGPNEVLLR	Intermediate Dimethyl (N-term)
DSG1	1352.7161	1352.7159	0.0002	36	MTGFELTEGVK	Dimethyl (K); Dimethyl (N-term)
	1360.7662	1360.7661	0.0001	43	MTGFELTEGVK	Intermediate Dimethyl (K); Intermediate Dimethyl (N-term)
AMDHD1	1206.6429	1206.6421	0.0008	21	SHTHGSLEVGK	Dimethyl (K); Dimethyl (N-term)
	1241.693	1241.6928	0.0002	29	SHTHGSLEVGK	Intermediate Dimethyl (K); Intermediate Dimethyl (N-term)
CDA	808.4403	808.44	0.0003	31	AVSEGYK	Dimethyl (K); Dimethyl (N-term)
	816.4904	816.4901	0.0003	34	AVSEGYK	Intermediate Dimethyl (K); Intermediate Dimethyl (N-term)
ALDH1A1	1571.7987	1571.7984	0.0003	59	TIPIDGNFFTYTR	Dimethyl (N-term)
	1575.8222	1575.8146	0.0077	52	TIPIDGNFFTYTR	Intermediate Dimethyl (N-term)
	1672.8348	1672.8348	0	55	IFVEESIYDEFVR	Dimethyl (N-term)
	1676.8612	1676.851	0.0102	60	IFVEESIYDEFVR	Intermediate Dimethyl (N-term)
	1769.9023	1769.9022	0.0002	74	LYSNAYLNDLAGCIK	Carbamidomethyl (C); Dimethyl (K); Dimethyl (N-term)
	1777.951	1777.9345	0.0165	29	LYSNAYLNDLAGCIK	Carbamidomethyl (C); Intermediate Dimethyl (K); Intermediate Dimethyl (N-term)
HBB	1341.6888	1341.6885	0.0003	37	VNVDEVGGEALGR	Dimethyl (N-term)
	1345.7138	1345.7134	0.0004	32	VNVDEVGGEALGR	Intermediate Dimethyl (N-term)
MFAP2	1501.7057	1501.7052	0.0005	26	QCLNEVCFYSLR	Carbamidomethyl (C); Dimethyl (N-term)
	1505.7307	1505.7305	0.0002	32	QCLNEVCFYSLR	Carbamidomethyl (C); Intermediate Dimethyl (N-term)
CBR1	1679.8957	1679.8955	0.0003	105	GQAAVQQLQAEGLSPR	Dimethyl (N-term)
	1683.922	1683.9116	0.0103	98	GQAAVQQLQAEGLSPR	Intermediate Dimethyl (N-term)
GAPDH	1557.8188	1557.8185	0.0003	81	VPTANVSVVDLTCR	Carbamidomethyl (C); Dimethyl (N-term)
	1561.844	1561.8347	0.0093	59	VPTANVSVVDLTCR	Carbamidomethyl (C); Intermediate Dimethyl (N-term)
	1640.9245	1640.925	-0.0004	64	LVINGNPITIFQER	Dimethyl (N-term)
	1644.9507	1644.9411	0.0095	55	LVINGNPITIFQER	Intermediate Dimethyl (N-term)
	1888.9743	1888.975	-0.0007	84	IISNASCTTNCLAPLAK	2 Carbamidomethyl (C); Dimethyl (K); Dimethyl (N-term)

	1897.0249	1897.0074	0.0175	84	IISNASCTTNCLAPLAK	2 Carbamidomethyl (C); Intermediate Dimethyl (K); Intermediate Dimethyl (N-term)
RHOG	1642.8535	1642.853	0.0005	38	TCLLICYTTNAFPK	Carbamidomethyl (C); Dimethyl (K); Dimethyl (N-term)
	1650.9036	1650.903	0.0006	34	TCLLICYTTNAFPK	Carbamidomethyl (C); Intermediate Dimethyl (K); Intermediate Dimethyl (N-term)
RAC1	1267.623	1267.6229	0.0001	37	YLECSALTQR	Carbamidomethyl (C); Dimethyl (N-term)
	1271.648	1271.6474	0.0006	30	YLECSALTQR	Carbamidomethyl (C); Intermediate Dimethyl (N-term)
LDHA	1161.5882	1161.5877	0.0005	32	VTLTSEEEAR	Dimethyl (N-term)
	1165.6124	1165.6039	0.0085	35	VTLTSEEEAR	Intermediate Dimethyl (N-term)
	1275.6236	1275.6241	-0.0005	53	VIGSGCNLDSAR	Carbamidomethyl (C); Dimethyl (N-term)
	1279.6491	1279.6403	0.0088	43	VIGSGCNLDSAR	Carbamidomethyl (C); Intermediate Dimethyl (N-term)
ABCA10	1492.7231	1492.7229	0.0002	47	CQDIVLEIDDFR	Carbamidomethyl (C); Dimethyl (N-term)
	1496.7481	1496.748	0.0001	45	CQDIVLEIDDFR	Carbamidomethyl (C); Intermediate Dimethyl (N-term)
PRDX6	933.4924	933.492	0.0005	25	NFDEILR	Dimethyl (N-term)
	937.5174	937.5081	0.0092	21	NFDEILR	Intermediate Dimethyl (N-term)
	1365.6598	1365.6599	-0.0001	36	DFTPVCTTELGR	Dimethyl (N-term)
	1422.6816	1422.6813	0.0003	45	DFTPVCTTELGR	Carbamidomethyl (C); Dimethyl (N-term)
	1426.706	1426.6975	0.0085	25	DFTPVCTTELGR	Carbamidomethyl (C); Intermediate Dimethyl (N-term)
TPI1	1261.6262	11261.626	0.0002	33	SNVSDAVAQSTR	Dimethyl (N-term)
	1265.6512	1265.6508	0.0004	31	SNVSDAVAQSTR	Intermediate Dimethyl (N-term)
TKT	1000.5803	1000.5804	-0.0001	25	IIALDGDTK	Dimethyl (K); Dimethyl (N-term)
	1008.6304	1008.6301	0.0003	28	IIALDGDTK	Intermediate Dimethyl (K); Intermediate Dimethyl (N-term)
AKR1B1	728.4051	728.4119	-0.0068	23	EVGAGIR	Intermediate Dimethyl (N-term)
	732.4301	732.4343	-0.0041	29	EVGAGIR	Intermediate Dimethyl (N-term)
PGK1	1124.644	1124.6441	-0.0001	23	VLPGVDALSNI	Dimethyl (N-term)
	1128.6695	1128.6603	0.0092	29	VLPGVDALSNI	Intermediate Dimethyl (N-term)
	1661.8163	1661.8162	0.0001	58	LGDVYVNDAFGTAHR	Dimethyl (N-term)
	1665.8428	1665.8324	0.0104	31	LGDVYVNDAFGTAHR	Intermediate Dimethyl (N-term)
GMPR	986.4933	986.493	0.0003	46	HAGGVAEYR	Dimethyl (N-term)
	990.5183	990.518	0.0003	44	HAGGVAEYR	Intermediate Dimethyl (N-term)
MFN2	1529.8387	1529.8383	0.0004	26	TVNQLAHALHQDK	Dimethyl (K); Dimethyl (N-term)
	1537.8888	1537.8881	0.0007	21	TVNQLAHALHQDK	Intermediate Dimethyl (K); Intermediate Dimethyl (N-term)

PCMT1	969.5605	969.5607	-0.0002	29	VQLVVGDGR	Dimethyl (N-term)
	973.5857	973.5769	0.0088	27	VQLVVGDGR	Intermediate Dimethyl (N-term)
IPO5	1685.9355	1685.9352	0.0003	53	AAVENLPTFLVELSR	Dimethyl (N-term)
	1689.9605	1689.9601	0.0004	50	AAVENLPTFLVELSR	Intermediate Dimethyl (N-term)
FNDC3A	1924.9894	1924.989	0.0004	38	VTSYIINNLQPDTTYR	Dimethyl (N-term)
	1929.0144	1929.0141	0.0003	31	VTSYIINNLQPDTTYR	Intermediate Dimethyl (N-term)
PRDM1	1818.984	1818.9835	0.0005	28	FGPLIGEIYTNDTVPK	Dimethyl (K); Dimethyl (N-term)
	1827.0341	1827.0339	0.0002	22	FGPLIGEIYTNDTVPK	Intermediate Dimethyl (K); Intermediate Dimethyl (N-term)
PARK7	782.5631	782.5629	0.0002	44	ALVILAK	Dimethyl (K); Dimethyl (N-term)
	790.6132	790.6129	0.0003	47	ALVILAK	Intermediate Dimethyl (K); Intermediate Dimethyl (N-term)
	901.5241	901.5233	0.0008	33	DGLILTSR	Dimethyl (N-term)
	901.5235	901.5233	0.0002	52	DGLILTSR	Dimethyl (N-term)
	905.5485	905.548	0.0005	41	DGLILTSR	Intermediate Dimethyl (N-term)
ALB	1538.8667	1538.8661	0.0006	37	VPQVSTPTLVEVSR	Dimethyl (N-term)
	1542.8917	1542.8912	0.0005	34	VPQVSTPTLVEVSR	Intermediate Dimethyl (N-term)
	1338.7659	1338.7653	0.0006	47	HPDYSVVLLLR	Dimethyl (N-term)
	1342.7909	1342.7908	0.0001	44	HPDYSVVLLLR	Intermediate Dimethyl (N-term)
BCORL1	1566.8002	1566.8001	0.0001	32	WQPDDVTESLPPK	Dimethyl (K); Dimethyl (N-term)
	1574.8503	1574.85	0.0003	29	WQPDDVTESLPPK	Intermediate Dimethyl (K); Intermediate Dimethyl (N-term)
PLCG1	558.3377	558.3377	0	30	AALEK	Dimethyl (K)
	566.3878	566.3876	0.0002	33	AALEK	Intermediate Dimethyl (N-term)
LSAMP	900.5027	900.5029	-0.0002	47	QGDTAILR	Dimethyl (N-term)
	904.5277	904.5272	0.0005	52	QGDTAILR	Intermediate Dimethyl (N-term)
HPD	1162.5618	1162.5612	0.0006	41	AFEEEQNLR	Dimethyl (N-term)
	1166.5868	1166.5864	0.0004	37	AFEEEQNLR	Intermediate Dimethyl (N-term)
MDH1	1420.735	1420.7348	0.0002	29	FVEGLPINDFSR	Dimethyl (N-term)
	1424.76	1424.7597	0.0003	27	FVEGLPINDFSR	Intermediate Dimethyl (N-term)
KRT6A	1029.6032	1029.6134	-0.0101	26	GEMALKDAK	Heavy Dimethyl (K); Intermediate Dimethyl (N-term)
	1073.6286	1073.6276	0.0011	22	GEMALKDAK	2 Intermediate Dimethyl (K); Intermediate Dimethyl (N-term); Oxidation (M)
BFSP1	904.4772	904.4767	0.0005	34	QLDAFQR	Dimethyl (N-term)

	908.5017	908.4928	0.0089	27	QLDAFQR	Intermediate Dimethyl (N-term)
	927.4808	927.4814	-0.0007	36	SYVFQTR	Dimethyl (N-term)
	931.5062	931.4976	0.0086	35	SYVFQTR	Intermediate Dimethyl (N-term)
	970.5446	970.5447	-0.0001	51	IIEIEGNR	Dimethyl (N-term)
	974.5692	974.5609	0.0083	47	IIEIEGNR	Intermediate Dimethyl (N-term)
	1207.6684	1207.6673	0.0011	57	EVLSHLQAQR	Dimethyl (N-term)
	1211.693	1211.6835	0.0095	33	EVLSHLQAQR	Intermediate Dimethyl (N-term)
	1307.6823	1307.6833	-0.001	62	EADEALLHNLR	Dimethyl (N-term)
	1311.7057	1311.6995	0.0062	55	EADEALLHNLR	Intermediate Dimethyl (N-term)
	1338.7138	1338.7143	-0.0004	75	LGELAGPEDALAR	Dimethyl (N-term)
	1342.7405	1342.7305	0.01	41	LGELAGPEDALAR	Intermediate Dimethyl (N-term)
	1582.8095	1582.8104	-0.0008	40	HVLVTGDANYVDPR	Dimethyl (N-term)
	1586.8357	1586.8265	0.0091	41	HVLVTGDANYVDPR	Intermediate Dimethyl (N-term)
	1727.9656	1727.9669	-0.0013	101	VELQAQTTTLEQAIK	Dimethyl (K); Dimethyl (N-term)
	1736.0158	1735.9992	0.0166	89	VELQAQTTTLEQAIK	Intermediate Dimethyl (K); Intermediate Dimethyl (N-term)
	1844.9879	1844.9883	-0.0005	62	LQLEAQFLQDDISAAK	Dimethyl (K); Dimethyl (N-term)
	1853.0373	1853.0207	0.0166	48	LQLEAQFLQDDISAAK	Intermediate Dimethyl (K); Intermediate Dimethyl (N-term)
LIM2	1385.7204	1385.72	0.0004	37	LSGSFAHQGLWR	Dimethyl (N-term)
	1389.7454	1389.7451	0.0003	33	LSGSFAHQGLWR	Intermediate Dimethyl (N-term)
SCNN1B	1690.7708	1690.7704	0.0004	26	NCNCGHYLYPLPR	Carbamidomethyl (C); Dimethyl (N-term)
	1694.7958	1694.7956	0.0002	22	NCNCGHYLYPLPR	Carbamidomethyl (C); Intermediate Dimethyl (N-term)
MIF	1445.7523	1445.752	0.0003	47	MPMFIVNTNVPR	Dimethyl (N-term)
	1449.7773	1449.7771	0.0002	41	MPMFIVNTNVPR	Intermediate Dimethyl (N-term)
ACTB	1003.4728	1003.4723	0.0005	54	AGFAGDDAPR	Dimethyl (N-term)
	1007.4972	1007.4885	0.0087	59	AGFAGDDAPR	Intermediate Dimethyl (N-term)
	1159.5512	1159.5509	0.0003	48	GYSFTTTAER	Dimethyl (N-term)
	1163.5756	1163.5671	0.0085	21	GYSFTTTAER	Intermediate Dimethyl (N-term)
	1543.7253	1543.7267	-0.0014	66	QEYDESGPSIVHR	Dimethyl (N-term)
	1547.7513	1547.7428	0.0085	34	QEYDESGPSIVHR	Intermediate Dimethyl (N-term)
	2009.1216	2009.1197	0.0019	44	VAPEEHPVLLTEAPLNPK	Dimethyl (K); Dimethyl (N-term)

	2017.1709	2017.152	0.0189	44	VAPEEHPVLLTEAPLNPK	Intermediate Dimethyl (K); Intermediate Dimethyl (N-term)
NME1	1371.7874	1371.7871	0.0003	33	TFIAIKPDGVQR	Dimethyl (N-term)
	1375.8124	1375.812	0.0004	29	TFIAIKPDGVQR	Intermediate Dimethyl (N-term)
RNF149	1391.7044	1391.7041	0.0003	27	NASAVVLYNEER	Dimethyl (N-term)
	1395.7294	1395.729	0.0004	26	NASAVVLYNEER	Intermediate Dimethyl (N-term)
ACTBL2	2009.1216	2009.1197	0.0019	32	VAPDEHPILLTEAPLNPK	Dimethyl (K); Dimethyl (N-term)
	2017.1709	2017.152	0.0189	33	VAPDEHPILLTEAPLNPK	Intermediate Dimethyl (K); Intermediate Dimethyl (N-term)
PRC1	1509.7133	1509.7132	0.0001	47	QTETEMLYGSAPR	Dimethyl (N-term)
	1513.7383	1513.7381	0.0002	44	QTETEMLYGSAPR	Intermediate Dimethyl (N-term)
KRT2	757.4333	757.4334	-0.0001	35	AAQAELK	Dimethyl (K)
	1004.5799	1004.5714	0.0084	21	IEISELNR	Intermediate Dimethyl (N-term)
	1384.7806	1384.7813	-0.0007	43	NLDLDSIIAEVK	Dimethyl (K); Dimethyl (N-term)
	1530.8406	1530.8406	0	58	FLEQQNQVLQTK	Dimethyl (K); Dimethyl (N-term)
	1538.8617	1538.8729	-0.0113	71	FLEQQNQVLQTK	Intermediate Dimethyl (K); Intermediate Dimethyl (N-term)
WIZ	1319.7521	1319.7518	0.0003	43	SPSDLHISPLAK	Dimethyl (K); Dimethyl (N-term)
	1327.8022	1327.802	0.0002	39	SPSDLHISPLAK	Intermediate Dimethyl (K); Intermediate Dimethyl (N-term)
	1111.5257	1111.5252	0.0005	28	SAGGEPGPEAGR	Dimethyl (N-term)
	1115.5507	1115.5503	0.0004	24	SAGGEPGPEAGR	Intermediate Dimethyl (N-term)
KRT10	1417.7077	1417.7048	0.0029	54	QSLEASLAETEGR	Dimethyl (N-term)
	1421.7327	1421.7316	0.0011	43	QSLEASLAETEGR	Intermediate Dimethyl (N-term)
DSP	1619.8036	1619.803	0.0006	35	LTEEETVCLDLDK	Carbamidomethyl (C); Dimethyl (K); Dimethyl (N-term)
	1627.8537	1627.8533	0.0004	37	LTEEETVCLDLDK	Carbamidomethyl (C); Intermediate Dimethyl (K); Intermediate Dimethyl (N-term)
KRT9	1087.5875	1087.587	0.0005	30	TLLDIDNTR	Dimethyl (K)
	1091.6125	1091.6035	0.009	27	TLLDIDNTR	Intermediate Dimethyl (N-term)
KRT14	1305.6095	1305.6094	0.001	37	GSCGIGGGIGGGSSR	Carbamidomethyl (C); Dimethyl (N-term)
	1309.6345	1309.6333	0.0012	32	GSCGIGGGIGGGSSR	Carbamidomethyl (C); Intermediate Dimethyl (N-term)
KRT1	1530.8406	1530.8406	0	58	FLEQQNQVLQTK	Dimethyl (K); Dimethyl (N-term)
	1538.8617	1538.8729	-0.0113	71	FLEQQNQVLQTK	Intermediate Dimethyl (K); Intermediate Dimethyl (N-term)
	1747.8983	1747.8913	0.007	62	QISNLQQSISDAEQR	Intermediate Dimethyl (N-term)
	1747.8998	1747.8913	0.0085	67	QISNLQQSISDAEQR	Intermediate Dimethyl (N-term)

PLA2R1	1621.7888	1621.788	0.0008	37	YGASWWIGLQEER	Dimethyl (N-term)
	1625.8138	1625.8131	0.0007	34	YGASWWIGLQEER	Intermediate Dimethyl (N-term)
COL4A1	1629.8798	1629.8791	0.0007	47	GDPGISGTPGAPGLPGPK	Dimethyl (K); Dimethyl (N-term)
	1637.9299	1637.929	0.0009	50	GDPGISGTPGAPGLPGPK	Intermediate Dimethyl (K); Intermediate Dimethyl (N-term)
	1396.6557	1396.6542	0.0015	33	GDPGFPGQPGMPGR	Dimethyl (N-term)
	1400.6807	1400.6687	0.0012	29	GDPGFPGQPGMPGR	Intermediate Dimethyl (N-term)
	953.5618	953.5612	0.0006	45	GQIGPIGEK	Dimethyl (K); Dimethyl (N-term)
	957.5868	957.5864	0.0004	43	GQIGPIGEK	Intermediate Dimethyl (K); Intermediate Dimethyl (N-term)
FBN1	1412.5877	1412.5875	0.0002	62	CDSGFALDSEER	Carbamidomethyl (C); Dimethyl (N-term)
	1416.6127	1416.6121	0.0006	59	CDSGFALDSEER	Carbamidomethyl (C); Intermediate Dimethyl (N-term)
KRT5	1221.5989	1221.5981	0.0008	34	YEELQQTAGR	Dimethyl (N-term)
	1225.6239	1225.6232	0.0007	31	YEELQQTAGR	Intermediate Dimethyl (N-term)
BFSP2	887.4615	887.4613	0.0001	33	SGNWGALR	Dimethyl (N-term)
	891.4862	891.4775	0.0087	38	SGNWGALR	Intermediate Dimethyl (N-term)
	1109.5134	1109.5141	-0.0007	43	YENEQPFR	Dimethyl (N-term)
	1113.5384	1113.5303	0.0081	28	YENEQPFR	Intermediate Dimethyl (N-term)
	1243.5788	1243.5793	-0.0004	67	AEAEQQQQER	Dimethyl (N-term)
	1247.6039	1247.5954	0.0084	56	AEAEQQQQER	Intermediate Dimethyl (N-term)
	1247.604	1247.5954	0.0085	45	AEAEQQQQER	Intermediate Dimethyl (N-term)
	1381.7562	1381.7565	-0.0003	64	SSGLATVPAPGLER	Dimethyl (N-term)
	1385.7812	1385.7727	0.0085	46	SSGLATVPAPGLER	Intermediate Dimethyl (N-term)
	1387.8199	1387.8187	0.0012	86	ALGISSVFLQGLR	Dimethyl (N-term)
	1391.8453	1391.8349	0.0104	60	ALGISSVFLQGLR	Intermediate Dimethyl (N-term)
	2089.9872	2089.9851	0.0021	102	ASWASSCQQVGEAVLENA	Carbamidomethyl (C); Dimethyl (N-term)
	2094.0128	2094.0013	0.0115	85	ASWASSCQQVGEAVLENA	Carbamidomethyl (C); Intermediate Dimethyl (N-term)
	2456.1959	2456.1965	-0.0006	57	VELHNTSCQVQSLQAETE	Carbamidomethyl (C); Dimethyl (N-term)
	2460.219	2460.2127	0.0062	34	VELHNTSCQVQSLQAETE	Carbamidomethyl (C); Intermediate Dimethyl (N-term)
PLEC	1212.6786	1212.6785	0.0001	29	AEAELLQQQK	Dimethyl (N-term)
	1188.648	1188.6562	-0.0082	26	AEAELLQQQK	Intermediate Dimethyl (N-term)
SPTAN1	1259.7335	1259.7337	-0.0002	53	DLSSVQTLLTK	Dimethyl (K); Dimethyl (N-term)

	1267.7836	1267.7831	0.0005	49	DLSSVQTLLTK	Intermediate Dimethyl (K); Intermediate Dimethyl (N-term)
	1351.7209	1351.7208	0.0001	30	SQLLGSAHEVQR	Dimethyl (N-term)
	1355.7459	1355.7451	0.0008	27	SQLLGSAHEVQR	Intermediate Dimethyl (N-term)
FABP5	1052.4781	1052.4774	0.0007	56	FEETTADGR	Dimethyl (N-term)
	1056.5023	1056.4936	0.0087	43	FEETTADGR	Intermediate Dimethyl (N-term)
	1326.6488	1326.649	-0.0002	60	TTQFSCTLGEK	Carbamidomethyl (C); Dimethyl (K); Dimethyl (N-term)
	1326.649	1326.649	0.0001	43	TTQFSCTLGEK	Carbamidomethyl (C); Dimethyl (K); Dimethyl (N-term)
	1052.476	1052.4774	-0.0014	44	FEETTADGR	Dimethyl (N-term)
	1056.5011	1056.4936	0.0075	56	FEETTADGR	Intermediate Dimethyl (N-term)
	1208.603	1208.6037	-0.0007	27	FEETTADGRK	Dimethyl (K); Dimethyl (N-term)
ALDOA	1673.8332	1673.8332	0	45	LQSIGTENTEENRR	Dimethyl (N-term)
	1677.8582	1677.858	0.0002	41	LQSIGTENTEENRR	Intermediate Dimethyl (N-term)
YWHAZ	1575.7378	1575.7376	0.0002	47	SVTEQGAELSNEER	Dimethyl (N-term)
	1579.7628	1579.7622	0.0006	40	SVTEQGAELSNEER	Intermediate Dimethyl (N-term)
PKM	2520.3473	2520.3475	-0.0002	30	TATESFASDPILYRPVAVAL	Dimethyl (K); Dimethyl (N-term)
	2528.3974	2528.3799	0.0175	31	TATESFASDPILYRPVAVAL	Intermediate Dimethyl (K); Intermediate Dimethyl (N-term)
PEBP1	2124.9806	2124.9826	-0.0021	74	APVAGTCYQAEWDDYVP	Carbamidomethyl (C); Dimethyl (K); Dimethyl (N-term)
	2133.0284	2133.015	0.0134	80	APVAGTCYQAEWDDYVP	Carbamidomethyl (C); Intermediate Dimethyl (K); Intermediate Dimethyl (N-term)
SPTBN1	1337.6589	1337.6583	0.0006	31	ALVADSHPESER	Dimethyl (N-term)
	1341.6839	1341.6737	0.0102	34	ALVADSHPESER	Intermediate Dimethyl (N-term)
S100A7	904.4312	904.4324	-0.0012	29	LMDDLDR	Dimethyl (N-term)
	908.4562	908.4486	0.0076	30	LMDDLDR	Intermediate Dimethyl (N-term)
	942.5129	942.5134	-0.0006	39	LQDAEIAR	Dimethyl (N-term)
	946.5377	946.5296	0.0081	43	LQDAEIAR	Intermediate Dimethyl (N-term)
S100AB	964.4855	964.4865	-0.001	39	TDEAAFQK	Dimethyl (K); Dimethyl (N-term)
	972.5356	972.5351	0.0005	42	TDEAAFQK	Intermediate Dimethyl (K); Intermediate Dimethyl (N-term)

Table S3. Dimethyl (regular formaldehyde) and intermediate (deuterated formaldehyde) labeled peptides identified from mouse lens ICAT samples by mass sectrometry (MS)

Protein I	pep_exp_n	pep_calc_r	pep_delt	pep	pep_seq	pep_var_mod
PARK7	782.5618	782.5629	-0.0011	47	ALVILAK	Dimethyl (K); Dimethyl (N-term)
	790.6119	790.6112	0.0007	36	ALVILAK	Intermediate Dimethyl (K); Intermediate Dimethyl (N-term)
	901.5217	901.5233	-0.0016	36	DGLILTSR	Dimethyl (N-term)
	905.5467	905.5462	0.0005	31	DGLILTSR	Intermediate Dimethyl (N-term)
EIF4A1	1167.6943	1167.6975	-0.0032	69	ATQALVLAPTR	Dimethyl (N-term)
	1171.7193	1171.7191	0.0002	72	ATQALVLAPTR	Intermediate Dimethyl (N-term)
SPTAN1	1318.6041	1318.6036	0.0005	57	DVEDEETWIR	Dimethyl (N-term)
	1322.6291	1322.6203	0.0089	51	DVEDEETWIR	Intermediate Dimethyl (N-term)
IPO5	1657.8485	1657.8477	0.0008	28	VAAAESMPLLLECAR	Carbamidomethyl (C); Dimethyl (N-term)
	1661.8735	1661.8693	0.0042	26	VAAAESMPLLLECAR	Carbamidomethyl (C); Intermediate Dimethyl (N-term)
EEF2	996.5695	996.5716	-0.0021	46	GGGQIIPTAR	Dimethyl (N-term)
	1000.5945	1000.5937	0.0008	42	GGGQIIPTAR	Intermediate Dimethyl (N-term)
TPI1	1274.62	1274.6215	-0.0015	57	SNVNDGVAQSTR	Dimethyl (N-term)
	1278.645	1278.6441	0.0009	62	SNVNDGVAQSTR	Intermediate Dimethyl (N-term)
GSS	1085.5697	1085.5717	-0.002	39	AVENELLDR	Dimethyl (N-term)
	1089.5954	1089.5878	0.0076	47	AVENELLDR	Intermediate Dimethyl (N-term)
	1760.0165	1760.0196	-0.0031	53	VGLLEALLPGQPEAVAR	Dimethyl (N-term)
	1764.0426	1764.0358	0.0069	21	VGLLEALLPGQPEAVAR	Intermediate Dimethyl (N-term)
FBLN1	1916.2437	1916.2433	0.0004	26	LVPLPLLLLSSLSLLAAR	Dimethyl (N-term)
	1920.2687	1920.2676	0.0011	22	LVPLPLLLLSSLSLLAAR	Intermediate Dimethyl (N-term)
RAC1	1584.8883	1584.8871	0.0012	27	HHCPNTPIILVGTK	Carbamidomethyl (C); Dimethyl (K); Dimethyl (N-term)
	1592.9384	1592.9372	0.0012	25	HHCPNTPIILVGTK	Carbamidomethyl (C); Intermediate Dimethyl (K); Intermediate Dimethyl (N-term)
ENO3	1494.7938	1494.797	-0.0032	52	YITPDQLADLYK	Dimethyl (K); Dimethyl (N-term)
	1502.844	1502.8293	0.0146	31	YITPDQLADLYK	Intermediate Dimethyl (K); Intermediate Dimethyl (N-term)
	1831.9635	1831.9679	-0.0045	73	AAVPSGASTGIYEALELR	Dimethyl (N-term)
	1835.9872	1835.9841	0.003	82	AAVPSGASTGIYEALELR	Intermediate Dimethyl (N-term)
ATP1B3	1036.6717	1036.6706	0.0011	34	QPLVAVQVK	Dimethyl (K); Dimethyl (N-term)

	1044.7218	1044.7212	0.0006	31	QPLVAVQVK	Intermediate Dimethyl (K); Intermediate Dimethyl (N-term)
TKT	896.5434	896.5443	-0.001	38	LAVSQVPR	Dimethyl (N-term)
	900.5679	900.5605	0.0074	27	LAVSQVPR	Intermediate Dimethyl (N-term)
	1678.82	1678.8178	0.0023	31	TVPFCSTFAAFFTR	Carbamidomethyl (C); Dimethyl (N-term)
	1682.8416	1682.834	0.0077	22	TVPFCSTFAAFFTR	Carbamidomethyl (C); Intermediate Dimethyl (N-term)
CLU	1715.8326	1715.8323	0.0003	35	VSTVTTHSSDSEVPSR	Dimethyl (N-term)
	1719.8576	1719.8577	-0.0001	31	VSTVTTHSSDSEVPSR	Intermediate Dimethyl (N-term)
GAPDH	1583.831	1583.8342	-0.0031	60	VPTPNVSVVDLTCR	Carbamidomethyl (C); Dimethyl (N-term)
	1587.8565	1587.8503	0.0062	44	VPTPNVSVVDLTCR	Carbamidomethyl (C); Intermediate Dimethyl (N-term)
	1874.956	1874.9594	-0.0034	96	IVSNASCTTNCLAPLAK	2 Carbamidomethyl (C); Dimethyl (K); Dimethyl (N-term)
	1883.0084	1882.9917	0.0167	79	IVSNASCTTNCLAPLAK	2 Carbamidomethyl (C); Intermediate Dimethyl (K); Intermediate Dimethyl (N-term)
MIF	742.4085	742.4081	0.0004	42	IGGAQNR	Dimethyl (N-term)
	746.4335	746.4323	0.0012	39	IGGAQNR	Intermediate Dimethyl (N-term)
CTSB	1408.6339	1408.6333	0.0006	31	HEAGDMMGGHAIR	Dimethyl (N-term)
	1412.6589	1412.6574	0.0015	29	HEAGDMMGGHAIR	Intermediate Dimethyl (N-term)
PHDGH	1126.6328	1126.6346	-0.0018	52	GGIVDEGALLR	Dimethyl (N-term)
	1130.6568	1130.6508	0.006	45	GGIVDEGALLR	Intermediate Dimethyl (N-term)
ASS1	1020.5951	1020.5968	-0.0017	27	YLLGTSLAR	Dimethyl (N-term)
	1024.6205	1024.6129	0.0075	25	YLLGTSLAR	Intermediate Dimethyl (N-term)
LIM2	759.3156	759.3142	0.0014	34	MHECR	Carbamidomethyl (C); Dimethyl (N-term)
	763.3406	763.3401	0.0005	31	MHECR	Carbamidomethyl (C); Intermediate Dimethyl (N-term)
	1385.7204	1385.7202	0.0002	53	LSGSFAHQGLWR	Dimethyl (N-term)
	1389.7454	1389.7453	0.0001	49	LSGSFAHQGLWR	Intermediate Dimethyl (N-term)
LGSN	897.5058	897.5072	-0.0014	29	VLPWAER	Dimethyl (N-term)
	901.5305	901.5234	0.0071	24	VLPWAER	Intermediate Dimethyl (N-term)
	900.5019	900.5029	-0.001	44	TGLQEVAR	Dimethyl (N-term)
	904.5264	904.519	0.0074	46	TGLQEVAR	Intermediate Dimethyl (N-term)
HSP90AB1	757.4697	757.4693	0.0004	35	LSELLR	Dimethyl (N-term)
	761.4947	761.4936	0.0011	31	LSELLR	Intermediate Dimethyl (N-term)
ALDH1A1	1216.6332	1216.6353	-0.0021	42	QAFQIGSPWR	Dimethyl (N-term)

	1220.6578	1220.6515	0.0064	44	QAFQIGSPWR	Intermediate Dimethyl (N-term)
LTBP2	1683.6174	1683.6171	0.0003	31	CEDVDECEGPQSSCR	Carbamidomethyl (C); Dimethyl (N-term)
	1687.6424	1687.6415	0.0009	29	CEDVDECEGPQSSCR	Carbamidomethyl (C); Intermediate Dimethyl (N-term)
UCHL1	950.5251	950.5259	-0.0008	27	FSAVALCK	Carbamidomethyl (C); Dimethyl (K); Dimethyl (N-term)
	958.5752	958.5749	0.0003	31	FSAVALCK	Carbamidomethyl (C); Intermediate Dimethyl (K); Intermediate Dimethyl (N-term)
	2010.9135	2010.9177	-0.0043	44	NEAIQAAHDSVAQEGQCR	Carbamidomethyl (C); Dimethyl (N-term)
	2014.9385	2014.9373	0.0012	34	NEAIQAAHDSVAQEGQCR	Carbamidomethyl (C); Intermediate Dimethyl (N-term)
COL4A2	1214.6017	1214.6044	-0.0027	56	GLDGFQGPSGPR	Dimethyl (N-term)
	1218.6267	1218.6264	0.0003	51	GLDGFQGPSGPR	Intermediate Dimethyl (N-term)
	1320.7484	1320.7514	-0.003	24	IAVQPGTLGPQGR	Dimethyl (N-term)
	1324.7734	1324.773	0.0004	27	IAVQPGTLGPQGR	Intermediate Dimethyl (N-term)
CCT4	795.4126	759.4121	0.0005	33	LTEYSR	Dimethyl (N-term)
	799.4376	799.4372	0.0004	30	LTEYSR	Intermediate Dimethyl (N-term)
MFAP2	1254.639	1254.6388	0.0002	27	TVCAHEELLR	Carbamidomethyl (C); Dimethyl (N-term)
	1258.664	1258.6631	0.0009	32	TVCAHEELLR	Carbamidomethyl (C); Intermediate Dimethyl (N-term)
HSPG2	1041.5809	1041.5802	0.0007	26	LGTVPQFPR	Dimethyl (N-term)
	1045.6059	1045.6133	-0.0074	24	LGTVPQFPR	Intermediate Dimethyl (N-term)
VIM	941.4808	941.4818	-0.001	41	SYVTTSTR	Dimethyl (N-term)
	945.5035	945.498	0.0055	32	SYVTTSTR	Intermediate Dimethyl (N-term)
	959.4901	959.4923	-0.0022	46	LLEGEESR	Dimethyl (N-term)
	963.5157	963.5085	0.0072	40	LLEGEESR	Intermediate Dimethyl (N-term)
	1120.5494	1120.5513	-0.0018	57	FADLSEAANR	Dimethyl (N-term)
	1124.5742	1124.5674	0.0068	39	FADLSEAANR	Intermediate Dimethyl (N-term)
	1142.5909	1142.5931	-0.0022	49	VELQELNDR	Dimethyl (N-term)
	1146.6159	1146.6093	0.0066	52	VELQELNDR	Intermediate Dimethyl (N-term)
	1281.5881	1281.5911	-0.003	57	LGDLYEEEMR	Dimethyl (N-term)
	1285.6131	1285.6072	0.0059	34	LGDLYEEEMR	Intermediate Dimethyl (N-term)
	1301.6084	1301.6022	0.0062	46	LGDLYEEEMR	Intermediate Dimethyl (N-term); Oxidation (M)
	1323.6286	1323.6306	-0.002	60	EEAESTLQSFR	Dimethyl (N-term)
	1327.6543	1327.6468	0.0075	54	EEAESTLQSFR	Intermediate Dimethyl (N-term)

	1364.6589	1364.6612	-0.0023	41	NLQEAEEWYK	Dimethyl (K); Dimethyl (N-term)
	1372.6899	1372.6935	-0.0036	32	NLQEAEEWYK	Intermediate Dimethyl (K); Intermediate Dimethyl (N-term)
	1471.7286	1471.7307	-0.0021	52	SLYSSSPGGAYVTR	Dimethyl (N-term)
	1475.7527	1475.7469	0.0058	47	SLYSSSPGGAYVTR	Intermediate Dimethyl (N-term)
	1545.8039	1545.8072	-0.0033	71	QVQSLTCEVDALK	Carbamidomethyl (C); Dimethyl (K); Dimethyl (N-term)
	1553.8543	1553.8396	0.0148	73	QVQSLTCEVDALK	Carbamidomethyl (C); Intermediate Dimethyl (K); Intermediate Dimethyl (N-term)
	1584.921	1584.9239	-0.0029	48	ISLPLPTFSSLNLR	Dimethyl (N-term)
	1588.9466	1588.9401	0.0066	34	ISLPLPTFSSLNLR	Intermediate Dimethyl (N-term)
	1863.8202	1863.8235	-0.0033	67	DGQVINETSQHHDDLE	Dimethyl (N-term)
	1867.8458	1867.8397	0.0061	60	DGQVINETSQHHDDLE	Intermediate Dimethyl (N-term)
MIXIP	1506.7136	1506.7131	0.0005	31	GMVSTSSLEEFHR	Dimethyl (N-term)
	1510.7386	1510.7382	0.0004	29	GMVSTSSLEEFHR	Intermediate Dimethyl (N-term)
HACE1	1862.956	1862.9551	0.0009	31	LFQTIVQMTQNEDLR	Dimethyl (N-term)
	1866.981	1866.9802	0.0008	33	LFQTIVQMTQNEDLR	Intermediate Dimethyl (N-term)
	997.5379	997.5374	0.0005	27	FIEFVCK	Carbamidomethyl (C); Dimethyl (K); Dimethyl (N-term)
	1005.588	1005.5872	0.0008	26	FIEFVCK	Carbamidomethyl (C); Intermediate Dimethyl (K); Intermediate Dimethyl (N-term)
ACTBL2	1817.9119	1817.9116	0.0003	71	SYELPDGQVITIGNER	Dimethyl (N-term)
	1821.9369	1821.9321	0.0048	67	SYELPDGQVITIGNER	Intermediate Dimethyl (N-term)
	2009.1178	2009.1197	-0.0019	35	VAPDEHPILLTEAPLNPK	Dimethyl (K); Dimethyl (N-term)
	2017.1665	2017.152	0.0145	35	VAPDEHPILLTEAPLNPK	Intermediate Dimethyl (K); Intermediate Dimethyl (N-term)
PAICS	1063.5086	1063.5085	0.0001	32	NFEWVADR	Dimethyl (N-term)
	1067.5336	1067.5332	0.0004	34	NFEWVADR	Intermediate Dimethyl (N-term)
TUBA1A	1514.8987	1514.9032	-0.0045	43	LISQIVSSITASLR	Dimethyl (N-term)
	1518.9273	1518.9193	0.008	77	LISQIVSSITASLR	Intermediate Dimethyl (N-term)
	1728.9276	1728.9298	-0.0022	66	AVFVDLEPTVIDEVR	Dimethyl (N-term)
	1732.9518	1732.946	0.0058	69	AVFVDLEPTVIDEVR	Intermediate Dimethyl (N-term)
	1745.9022	1745.906	-0.0038	28	NLDIERPTYTNLNR	Dimethyl (N-term)
	1749.928	1749.9222	0.0058	29	NLDIERPTYTNLNR	Intermediate Dimethyl (N-term)
	2436.2289	2436.2325	-0.0037	86	FDGALNVDLTEFQTNLVPYPR	Dimethyl (N-term)
	2440.2489	2440.2487	0.0002	38	FDGALNVDLTEFQTNLVPYPR	Intermediate Dimethyl (N-term)

IDH1	771.4239	771.4235	0.0004	29	SPNGTIR	Dimethyl (N-term)
	775.4489	775.4481	0.0008	25	SPNGTIR	Intermediate Dimethyl (N-term)
	1368.6997	1368.6991	0.0006	34	TVEAEAAHGTVTR	Dimethyl (N-term)
	1372.7247	1372.7242	0.0005	37	TVEAEAAHGTVTR	Intermediate Dimethyl (N-term)
ACTB	972.5742	972.5757	-0.0015	46	AVFPSIVGR	Dimethyl (N-term)
	976.5987	976.5918	0.0069	37	AVFPSIVGR	Intermediate Dimethyl (N-term)
	1003.4709	1003.4723	-0.0014	61	AGFAGDDAPR	Dimethyl (N-term)
	1007.4961	1007.4885	0.0076	57	AGFAGDDAPR	Intermediate Dimethyl (N-term)
	1225.7274	1225.7295	-0.0022	30	AVFPSIVGRPR	Dimethyl (N-term)
	1229.7524	1229.7457	0.0067	29	AVFPSIVGRPR	Intermediate Dimethyl (N-term)
	2009.1178	2009.1197	-0.0019	47	VAPEEHPVLLTEAPLNPK	Dimethyl (K); Dimethyl (N-term)
	2017.1665	2017.152	0.0145	51	VAPEEHPVLLTEAPLNPK	Intermediate Dimethyl (K); Intermediate Dimethyl (N-term)
NDUFA11	1371.7511	1371.7504	0.0007	28	LEGWELFPTPK	Dimethyl (K); Dimethyl (N-term)
	1379.8012	1379.8001	0.0011	27	LEGWELFPTPK	Intermediate Dimethyl (K); Intermediate Dimethyl (N-term)
BFSP1	826.5015	826.5025	-0.001	38	VAVQVQR	Dimethyl (N-term)
	830.5261	830.5187	0.0074	34	VAVQVQR	Intermediate Dimethyl (N-term)
	897.4691	897.4708	-0.0017	37	SYVFQAR	Dimethyl (N-term)
	901.4936	901.487	0.0066	26	SYVFQAR	Intermediate Dimethyl (N-term)
	904.4756	904.4767	-0.0011	23	QLDAFQR	Dimethyl (N-term)
	908.5006	908.4928	0.0078	24	QLDAFQR	Intermediate Dimethyl (N-term)
	943.5319	943.5338	-0.0019	32	IIEIEGSR	Dimethyl (N-term)
	947.5573	947.55	0.0074	30	IIEIEGSR	Intermediate Dimethyl (N-term)
	1154.6745	1154.6771	-0.0026	49	QLAVAQQTLR	Dimethyl (N-term)
	1154.675	1154.6771	-0.0021	57	QLAVAQQTLR	Dimethyl (N-term)
	1158.7002	1158.6933	0.0069	40	QLAVAQQTLR	Intermediate Dimethyl (N-term)
	1379.7031	1379.7045	-0.0014	79	LGEQPGPEDALAR	Dimethyl (N-term)
	1383.7284	1383.7206	0.0077	72	LGEQPGPEDALAR	Intermediate Dimethyl (N-term)
	1483.7602	1483.763	-0.0028	76	EVAALQNQLEEGR	Dimethyl (N-term)
	1487.7854	1487.7792	0.0062	71	EVAALQNQLEEGR	Intermediate Dimethyl (N-term)
	1669.922	1669.925	-0.003	112	AELQAQTTALEQAIK	Dimethyl (K); Dimethyl (N-term)

	1677.9721	1677.9574	0.0148	87	AELQAQTTALEQAIK	Intermediate Dimethyl (K); Intermediate Dimethyl (N-term)
HTRA1	1407.8237	1407.8223	0.0014	52	IAPAVVHIELYR	Dimethyl (N-term)
	1411.8487	1411.8472	0.0015	49	IAPAVVHIELYR	Intermediate Dimethyl (N-term)
FABP5	1038.4601	1038.4618	-0.0018	45	FDETTADGR	Dimethyl (N-term)
	1042.4852	1042.478	0.0072	35	FDETTADGR	Intermediate Dimethyl (N-term)
	1194.5865	1194.5881	-0.0015	35	FDETTADGRK	Dimethyl (K); Dimethyl (N-term)
COL4A4	811.3824	811.3812	0.0012	31	GEPGPDGR	Dimethyl (N-term)
	815.4074	815.4061	0.0013	33	GEPGPDGR	Intermediate Dimethyl (N-term)
	1471.8583	1471.8581	0.0002	57	GPLGSPGLNGLHGLK	Dimethyl (K); Dimethyl (N-term)
	1479.9084	1479.9081	0.0003	62	GPLGSPGLNGLHGLK	Intermediate Dimethyl (K); Intermediate Dimethyl (N-term)
ALB	1466.8064	1466.8093	-0.0029	40	APQVSTPTLVEAAR	Dimethyl (N-term)
	1470.8323	1470.8255	0.0068	27	APQVSTPTLVEAAR	Intermediate Dimethyl (N-term)
	1736.8932	1736.8985	-0.0053	20	LSQTFPNADFAEITK	Dimethyl (K); Dimethyl (N-term)
	1744.9466	1744.9308	0.0158	22	LSQTFPNADFAEITK	Intermediate Dimethyl (K); Intermediate Dimethyl (N-term)
VTN	817.4116	817.4109	0.0007	34	VDAAMAGR	Dimethyl (N-term)
	821.4366	821.436	0.0006	37	VDAAMAGR	Intermediate Dimethyl (N-term)
PFN1	1509.8166	1509.8191	-0.0025	35	SSFFVNGLTLGGQK	Dimethyl (K); Dimethyl (N-term)
	1517.8667	1517.8662	0.0005	31	SSFFVNGLTLGGQK	Intermediate Dimethyl (K); Intermediate Dimethyl (N-term)
PKM	867.5525	867.5542	-0.0016	42	APIIAVTR	Dimethyl (N-term)
	871.5775	871.5703	0.0072	40	APIIAVTR	Intermediate Dimethyl (N-term)
	1386.7269	1386.7289	-0.002	56	NTGIICTIGPASR	Carbamidomethyl (C); Dimethyl (N-term)
	1390.7508	1390.7451	0.0057	56	NTGIICTIGPASR	Carbamidomethyl (C); Intermediate Dimethyl (N-term)
	2548.3454	2548.3424	0.003	21	EATESFASDPILYRPVAVALDTK	Dimethyl (K); Dimethyl (N-term)
	2556.3909	2556.3748	0.0161	25	EATESFASDPILYRPVAVALDTK	Intermediate Dimethyl (K); Intermediate Dimethyl (N-term)
NID1	1203.6585	1203.6611	-0.0026	44	YALSNSIGPVR	Dimethyl (N-term)
	1207.6837	1207.6773	0.0064	41	YALSNSIGPVR	Intermediate Dimethyl (N-term)

Figure S1. To quantitatively determine cysteine disulfide bond formation by Isotope-coded affinity tag (ICAT) and dimethyl labeling proteomics approach

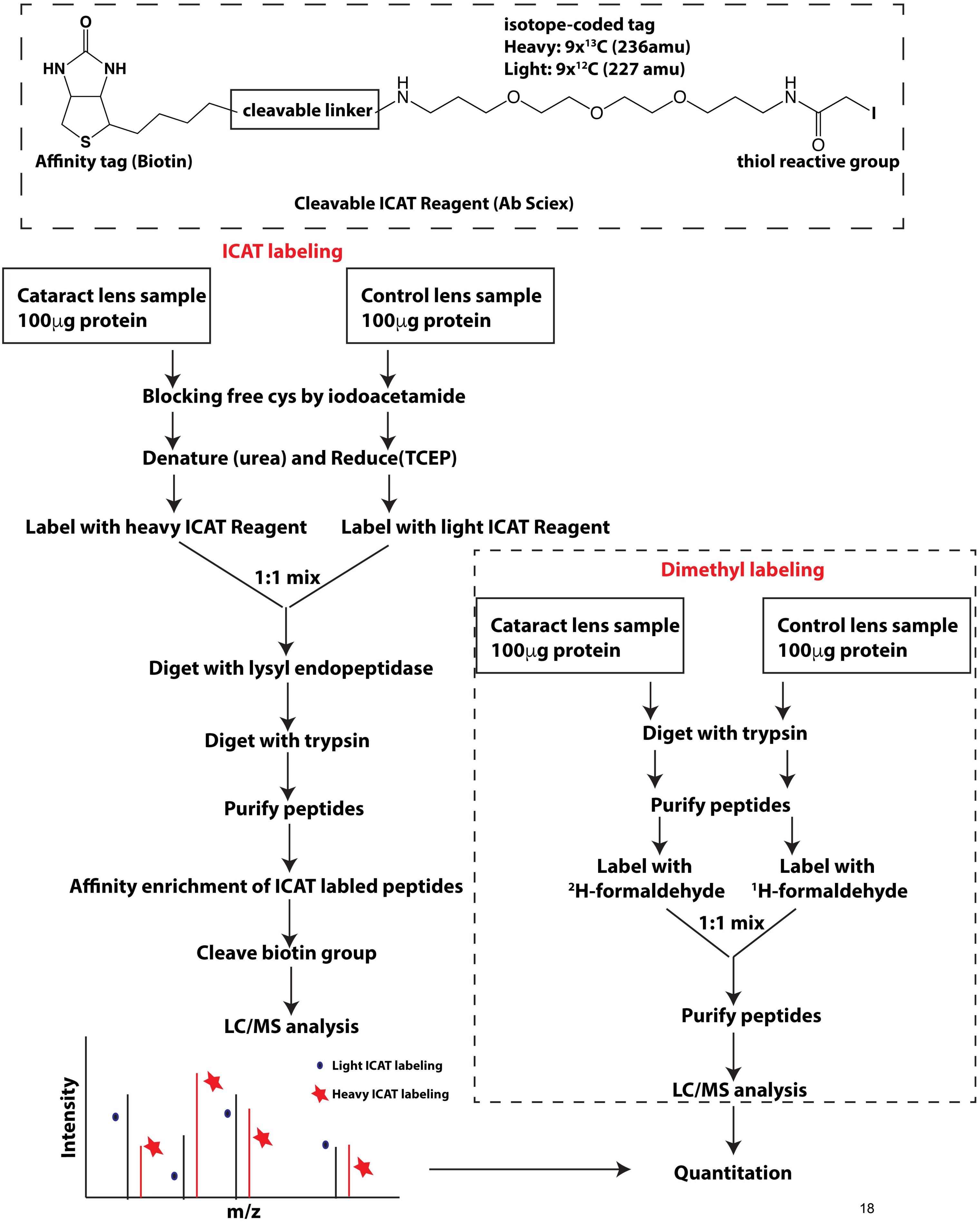

Figure S2

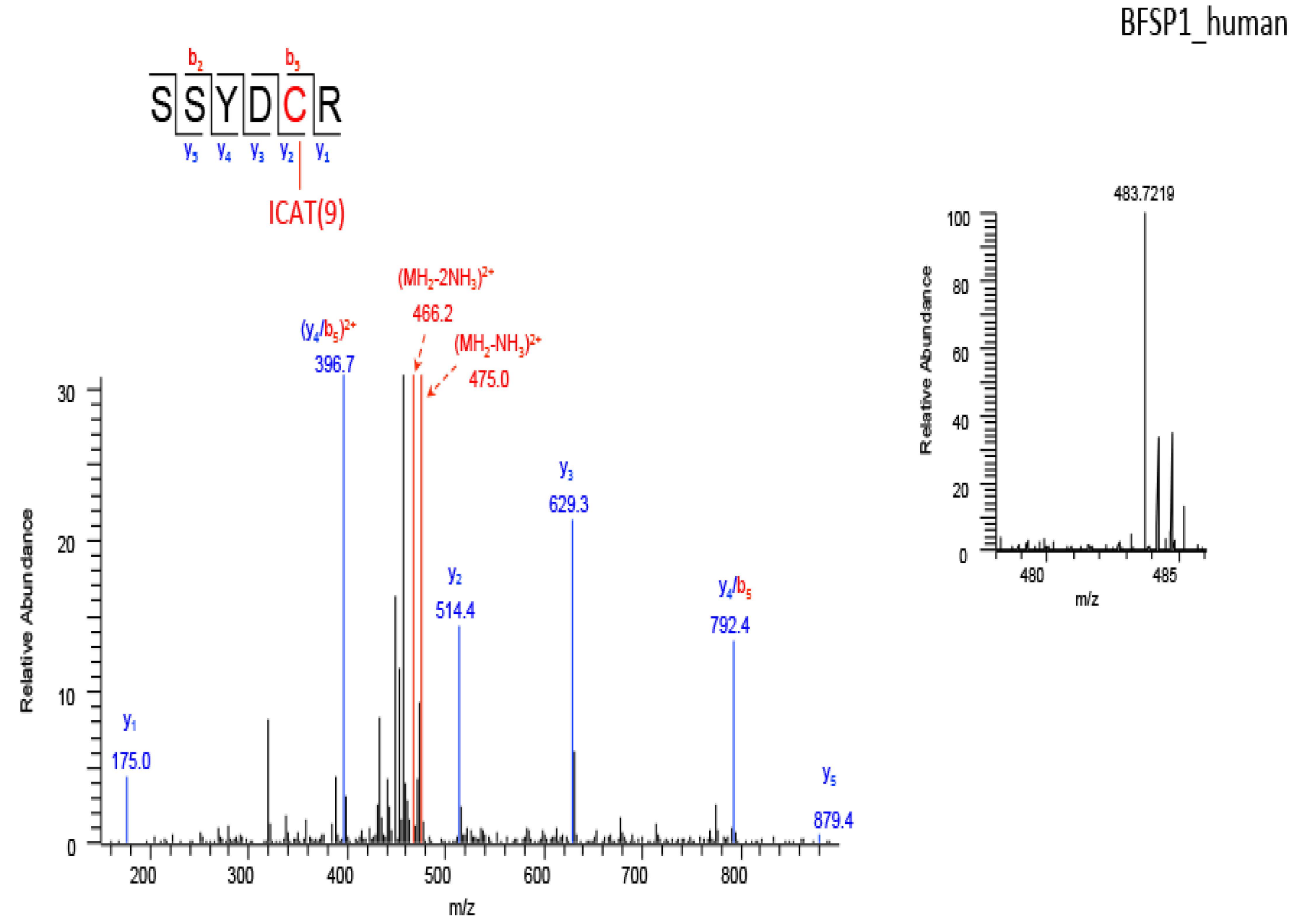

Tandem MS (MS2) of peptide SSYDCR of BFSP1 protein with heavy ICAT(9) labelling. The series y ions and b ions clearly show that the modification is at Cys 292 residue. The insert window shows MS1 of this peptide with m/z of 483.7219.

Figure S2. Representative mass spectrum of ICAT labeling.

Figure S3

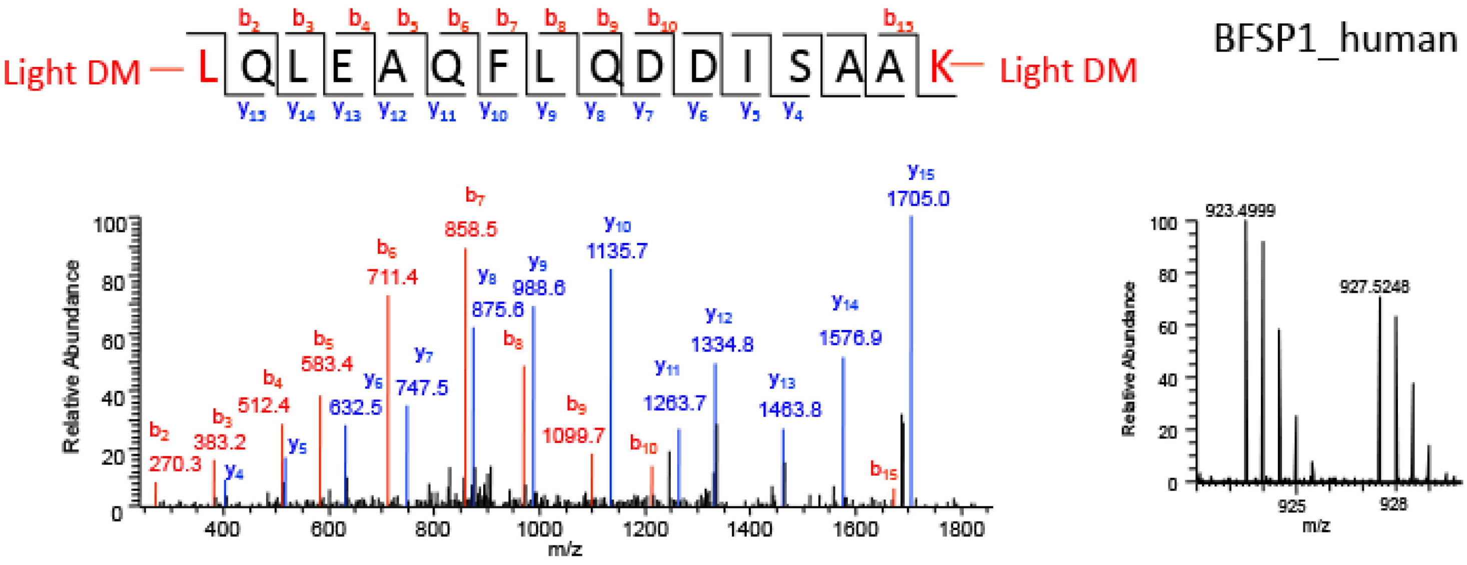

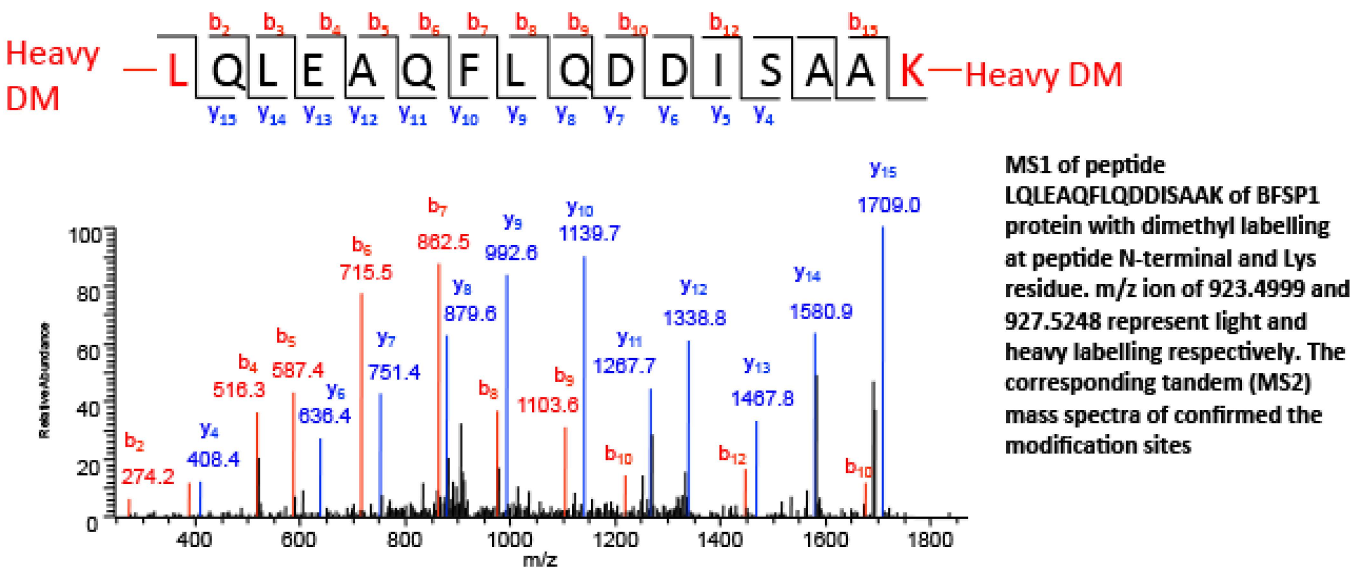

MS1 of peptide LQLEAQFLQDDISAAK of BFSP1 protein with dimethyl labelling at peptide N-terminal and Lys residue. m/z ion of 923.4999 and 927.5248 represent light and heavy labelling respectively. The corresponding tandem (MS2) mass spectra of confirmed the modification sites

Figure S3. Representative mass spectrum of dimethyl labeling. Human BFSP1 peptide LQLEAQFLQDDISAAK light, heavy dimethyl labeling spectrum and their m/z identification were shown in top and bottom panel. The ratio between heavy and light labeling was shown in right panel.
